# Molecular neuroimaging of Parkinson's disease: association of motor and non-motor symptoms with synaptic density, dopaminergic and serotonergic systems

**DOI:** 10.1016/j.ynirp.2026.100337

**Published:** 2026-03-31

**Authors:** Julia J. Schubert, Silvia Rota, Lucia Batzu, Pavlos Zinzalias, Nazneen Barwick, Sarah Lee, Mattia Veronese, Alastair D. Reith, Christine A. Parker, Steve C.R. Williams

**Affiliations:** a Department of Neuroimaging, Institute of Psychiatry, Psychology & Neuroscience, King's College London, London, UK; b Department of Basic and Clinical Neurosciences, Institute of Psychiatry, Psychology and Neuroscience, King's College London, London, UK; c Parkinson's Foundation Centre of Excellence, King's College Hospital, London, UK; d Emerging Therapies Department, GSK, Stevenage, UK; e Clinical Imaging Group, GSK, Stevenage, UK; f Department of Information Engineering, University of Padua, Padua, Italy; g Novel Human Genetics Research Unit, GSK, Stevenage, UK

**Keywords:** Parkinson's disease, Molecular imaging, Dopamine, Serotonin, Synaptic density

## Abstract

Parkinson's disease (PD) is a neurodegenerative disease characterised by molecular and structural brain changes detectable through advanced imaging. Understanding alterations in neurotransmitter systems and synaptic density, and their clinical relevance, is critical for identifying disease-specific biomarkers and therapeutic targets. This study included 33 PD patients (27 idiopathic PD (iPD) and 6 LRRK2 mutation carriers) (5.2 ± 3.6 years from diagnosis, 2.1 ± 0.7 Hoehn & Yahr OFF state) and 25 healthy controls (HC). Longitudinal data were collected for 20 iPD and 22 HC (10-33 months post-baseline; 20.2 ± 7.3 months). Participants underwent clinical assessments, structural magnetic resonance imaging, ^11^C-UCB-J positron emission tomography (PET) to assess synaptic density, ^11^C-DASB PET to assess serotonin transporter density, and ^123^I-FP-CIT single-photon emission computed tomography to assess dopamine transporter density. Analyses included baseline group comparisons, clinical correlations, and longitudinal assessments. At baseline, lower ^123^I-FP-CIT uptake in caudate and putamen (p < 0.001) and reduced ^11^C-DASB binding in the insular cortex (p = 0.003), parietal lobe (p = 0.009), caudate (p < 0.001), and putamen (p = 0.002) were observed in PD compared to HC. Some baseline correlations emerged between imaging metrics and symptom scales in PD, though these were limited. Despite progression in motor impairment, autonomic dysfunction, and overall disability in PD, no significant longitudinal changes or group × time interactions were detected for molecular imaging measures. This study confirmed dopaminergic and serotonergic dysfunction in PD. Synaptic density did not differ between groups or change over time, suggesting synaptic loss may be minimal at mild-to-moderate disease stages. These findings highlight how different molecular imaging markers reflect distinct aspects and timescales of PD pathophysiology.

## Highlights

• Dopaminergic and serotonergic dysfunction confirmed in mild-to-moderate Parkinson's disease.
• No significant group differences or longitudinal change in synaptic density detected using ^11^C-UCB-J PET.
• Limited overlap between molecular imaging changes and clinical progression over ∼20 months.
• Imaging–symptom associations suggest distinct dopaminergic, serotonergic, and synaptic contributions to PD heterogeneity.

## 1 Introduction

Parkinson's Disease (PD) is the second most common neurodegenerative disorder, primarily characterized by the progressive loss of dopaminergic neurons in the substantia nigra (SN) and striatal terminals. The presence of Lewy bodies, constituted by α-synuclein aggregates, is the hallmark of PD (Schapira and Jenner, 2011), and its pathogenesis generally involves a distinct neuropathological progression, from brainstem regions to cortical areas (Braak et al., 2003). Recent studies of in vivo synaptic density seem to support this hypothesis, showing that synaptic density is first reduced in the SN, and then into the cortical regions as the disease progresses, for example with the development of severe cognitive impairment (Andersen et al., 2021; Holmes et al., 2024). Besides dopamine, other neurotransmitter systems are involved in the pathogenesis of PD, including glutamatergic, serotonergic, GABAergic, histaminergic, adrenergic, and cholinergic pathways within the cortex, brainstem, and basal ganglia (Freitas and Fox, 2016), which represent potential biomarkers and therapeutic targets. Typically sporadic, familial cases account for 10-15% of PD cases (Wider and Wszolek, 2007). Among these, leucine-rich repeat kinase 2 (LRRK2)-associated PD (LRRK2-PD) generally resembles idiopathic PD (iPD) in clinical presentation and, in most cases, pathology. However, LRRK2-PD exhibits some distinct clinical features, such as a lower risk of cognitive decline (Alcalay et al., 2013; Ross et al., 2006).

Molecular imaging using positron emission tomography (PET) and single-photon emission computed tomography (SPECT) has significantly advanced the understanding of PD. Imaging the dopamine transporter (DAT) has consistently revealed a reduced striatal uptake in both iPD and LRRK2-PD, indicative of dopaminergic loss (Marras et al., 2016). Serotonin transporter (SERT) dysfunction, identified through ^11^C-DASB PET, is evident in PD and linked to non-motor symptoms, such as depression and dyskinesias (Pagano et al., 2017). Synaptic loss, assessed through the PET ligand ^11^C-UCB-J targeting synaptic vesicle protein 2A (SV2A), is increasingly recognized as a crucial aspect of PD pathophysiology, particularly in regions such as the SN and locus coeruleus (Holmes et al., 2024; Delva et al., 2020; Matuskey et al., 2020; Martin et al., 2023). However, one study found no significant ^11^C-UCB-J uptake differences in SN or locus coeruleus, but noted reductions in other regions including the caudate, putamen, thalamus, brainstem, dorsal raphe, and cortical regions in drug-naïve PD (Wilson et al., 2020). While cross-sectional studies have consistently found synaptic density loss in PD, including but not exclusively in the SN, longitudinal analyses have not demonstrated significant changes in synaptic density over time in PD populations (Delva et al., 2020; Wilson et al., 2020).

Given these findings, this study aimed to investigate differences in three molecular imaging biomarkers (dopaminergic, serotonergic, and synaptic density), between PD patients in mild-to-moderate stages and matched healthy controls (HC), in both a cross-sectional and longitudinal setting. Each molecular marker offers a different insight into the PD pathology, thereby capturing a more comprehensive view of the disease mechanisms. This research also sought to elucidate if any molecular alterations were able to correlate with clinical features of PD, and if feasible, highlight potential therapeutic targets and mechanisms that may inform future treatment strategies.

## 2 Methods

### 2.1 Participants

Participants were recruited as part of the *Longitudinal Study of Molecular Pathology and Neuronal Networks in Leucine-rich Repeat Kinase 2 Carriers and Idiopathic Parkinson's Disease Patients and Healthy Controls Using PET, MR Imaging, and Other Markers of in Vivo Pathology* (NCT03782753). The study was authorised by local ethics committees (NRES South East London REC, IRAS 249061, REC reference (Ashburner, 2007)/LO/1915). PD patients were recruited from the Parkinson's Foundation Centre of Excellence at King's College Hospital, London, UK. 25 HC and PD patients, comprising 27 iPD and 6 LRRK2-PD, were enrolled at baseline. A subset of 22 HC and 20 iPD also received follow-up assessments between 10- and 33-months post-baseline (mean: 20.2 ± 7.3 months). Demographic details are summarised in Table 1, Table 2.

**Table 1:** Baseline demographic and clinical characteristics.

Variable mean (SD)	Parkinson's			Healthy Controls	*p*	
	All n = 33	iPD n = 27	LRRK2-PD n = 6	n = 25	HCvsAll	HCvsiPD
Age, years	60.5 (9.5)	60.5 (9.1)	60.7 (12.2)	61.3 (8.7)	0.752	0.748
Male, n (%)	21 (64%)	19 (70%)	2 (33%)	19 (76%)	0.313	0.647
BMI, kg/m2	27.0 (5.0)	26.6 (4.2)	28.8 (7.8)	26.4 (4.2)	0.919	0.934
Education, years	15.8 (3.6)	16.4 (3.2)	13.2 (4.2)	16.6 (3.1)	0.372	0.797
PD duration, years	5.2 (3.6)	4.6 (3.2)	7.7 (4.4)	-	-	-
MDS-UPDRS total	52.3 (21.7)	49.0 (21.1)	67.0 (20.0)	1.8 (1.3)	<0.001∗	<0.001∗
MDS-UPDRS III	31.8 (13.0)	30.2 (12.3)	38.7 (15.0)	0.3 (1.0)	<0.001∗	<0.001∗
SCOPA-AUT	10.5 (7.3)	10.1 (7.6)	12.7 (6.2)	4.5 (4.0)	<0.001∗	<0.001∗
BDI	8.4 (6.4)	8.2 (6.9)	9.2 (3.6)	2.2 (3.1)	<0.001∗	<0.001∗
MoCA	27.2 (2.2)	27.8 (1.8)	24.5 (2.1)	28.4 (1.4)	0.036∗	0.217
Hoehn & Yahr off	2.1 (0.7)	2.0 (0.6)	2.7 (1.0)	-	-	-
LEDD total, mg	520.0 (340.9)	454.9 (273.6)	862.3 (437.9)	-	-	-

∗ Significant difference *p* < 0.05, note values shown are mean (standard deviation; SD) unless otherwise indicated. Group differences were evaluated for all Parkinson's disease (PD) participants versus healthy controls (HCvsAll) and for iPD patients versus healthy controls (HCvsiPD). Disease duration was reported in years since diagnosis. BDI = Beck Depression Inventory; BMI = Body Mass Index; LEDD = Levodopa Equivalent Daily Dose; MDS-UPDRS = Movement Disorder Society–Unified PD Rating Scale; MDS-UPDRS III = motor examination subscale of the MDS-UPDRS; MoCA = Montreal Cognitive Assessment; SCOPA-AUT = Scales for Outcomes in PD–Autonomic.

**Table 2:** Demographic and clinical characteristics from longitudinal subset of subjects.

Variable mean (SD)	Baseline Subset			Follow-up			
	Parkinson's	Healthy Controls	*p*	Parkinson's	Healthy Controls	*p*	
	n = 20	n = 22	HCvsPD	n = 20	n = 22	HCvsPD	BLvsFOL
Age, years	61.6 (8.8)	61.5 (7.7)	0.983	63.5 (8.6)	62.5 (7.1)	0.870	-
Male, n (%)	15 (75%)	17 (77%)	0.863	15 (75%)	17 (77%)	0.863	-
BMI, kg/m2	25.8 (3.6)	26.1 (4.0)	0.837	25.5 (3.9)	26.3 (4.4)	0.597	0.281
PD duration, years	4.8 (3.2)	-	-	6.5 (3.4)	-	-	-
MDS-UPDRS III	30.0 (11.7)	0.4 (1.0)	<0.001∗	34.8 (11.7)	0.2 (0.6)	<0.001∗	0.013∗
MDS-UPDRS total	44.9 (17.8)	1.9 (1.3)	<0.001∗	52.8 (20.2)	2.8 (2.4)	<0.001∗	0.023∗
MoCA	28.0 (1.7)	28.5 (1.4)	0.381	29.0 (0.9)	28.5 (1.4)	0.347	0.004∗
SCOPA-AUT	7.2 (3.5)	4.2 (3.6)	0.008∗	9.2 (4.6)	4.9 (3.5)	0.003∗	0.006∗
BDI	6.5 (5.8)	2.4 (3.2)	0.003∗	6.3 (5.2)	2.0 (3.5)	<0.001∗	0.285
Hoehn & Yahr off	1.9 (0.6)	-	-	2.2 (0.4)	-	-	0.886
LEDD total, mg	401.8 (256.2)	-	-	546.0 (275.3)	-	-	0.002∗

∗ Significant difference *p* < 0.05, note values shown are mean (standard deviation; SD) unless otherwise indicated. Baseline versus follow-up (BLvsFOL) p-values refer only to Parkinson's disease (PD) subjects that had both baseline and follow-up assessments. Group differences were evaluated for PD participants versus healthy controls (HCvsPD) that had both baseline and follow-up assessments. Disease duration was reported in years since diagnosis. BDI = Beck Depression Inventory; BMI = Body Mass Index; LEDD = Levodopa Equivalent Daily Dose; MDS-UPDRS = Movement Disorder Society–Unified PD Rating Scale; MDS-UPDRS III = motor examination subscale of the MDS-UPDRS; MoCA = Montreal Cognitive Assessment; SCOPA-AUT = Scales for Outcomes in PD–Autonomic.

Participants were aged 30-85 years and provided signed, informed consent before any study procedures. They were required to demonstrate adequate visual and auditory acuity to complete psychological testing and had to comply with study restrictions, attend all evaluations, and complete all required tests and procedures. The LRRK2-PD group included patients with genetically confirmed LRRK2 mutations, clinically verified prior to study start. Both LRRK2-PD and iPD subjects were diagnosed after age 30 and met the Movement Disorder Society Clinical Diagnostic Criteria for PD and classified as Hoehn and Yahr (H&Y) stage 1-3 in the ON state, whether drug-naïve or on dopamine replacement therapy.

Participants were excluded if they had significant medical conditions, recent infections, a history of drug or alcohol abuse, neurological disorders, contraindications to magnetic resonance imaging (MRI), recent cancer, or if they were pregnant or breastfeeding. Those taking serotonin-affecting drugs, SV2A-targeting medications, antipsychotics, corticosteroids, or other prohibited drugs within specified timeframes were also excluded. Exclusion criteria also included any advanced PD treatments available at the time of the study, such as apomorphine subcutaneous infusion, levodopa/carbidopa intrajejunal infusion, and deep brain stimulation. Additional exclusions were recent changes in PD medication, and radiation exposure exceeding 10 mSv within the past year.

This study originally aimed to characterise the molecular mechanisms underlying LRRK2 Parkinsonism to gain deeper insights into potential pathways in PD and identify potential targets for disease-modifying therapies. However, COVID-19 restrictions significantly affected recruitment and travel, leading to an insufficient number of LRRK2-PD participants for independent group comparisons and absence of all LRRK2-PD participants at follow-up. Baseline data collected from the LRRK2-PD group is therefore presented both in combination with iPD data and qualitatively as an independent group, as sample sizes do not support statistical comparisons across iPD, LRRK2-PD, and HC groups.

The study originally planned for a 12-month follow-up period. However, due to COVID-19-related amendments, the follow-up window was extended, with actual follow-up imaging assessments ranging from 10- to 33-months post-baseline (mean: 20.2 ± 7.3 months). Participants newly enrolled after the protocol amendment were followed for at least 12 months, while those already enrolled underwent follow-up at approximately 12- or 24-months, depending on their stage in the study. To maximize statistical power and account for variability in follow-up timing, all follow-up data were pooled for longitudinal analyses, with time since baseline accounted for in the statistical methods.

### 2.2 Clinical assessments

Participants underwent a series of clinical assessments, including the Movement Disorder Society-Unified Parkinson's Disease Rating Scale (MDS-UPDRS) and H&Y staging. Autonomic dysfunction was assessed using the Scales for Outcomes in Parkinson's Disease-Autonomic (SCOPA-AUT), a 26-item scale, while sleep quality was measured with the Parkinson's Disease Sleep Scale (PDSS), which addresses common sleep disturbances in PD (Chaudhuri et al., 2002). Daytime sleepiness was quantified using the Epworth Sleepiness Scale (ESS), a brief eight-item self-report scale. Symptoms of REM sleep behaviour disorder (RBD) were evaluated with the RBD Screening Questionnaire (RBDSQ), capturing symptoms such as vivid dreaming and nocturnal movements. Additional assessments included the Modified Constipation Assessment Scale (MCAS) for evaluating constipation severity, the PD Fatigue Scale (PFS-16) for fatigue, the King's Parkinson's Disease Pain Scale (KPPS), the University of Pennsylvania Smell Identification Test (UPSIT) to assess olfactory function, and the Beck's Depression Inventory (BDI) and Apathy Evaluation Scale (AES) to assess depression and apathy, respectively.

Cognitive function was evaluated using the Montreal Cognitive Assessment (MoCA), well-known for assessing cognitive decline. Executive functions and cognitive processing speed, as well as working memory and cognitive flexibility were further assessed with the Symbol Digit Modalities Test (SDMT) and the Letter-Number Sequencing Test (LNSI), respectively.

UPSIT was not collected for three PD at baseline and AES for one PD at baseline and one PD at follow-up.

### 2.3 PET and MRI data acquisition

MRI was performed for all subjects at baseline (25 HC and 33 PD) and follow-up (22 HC and 20 PD) using a structural 3D T1-weighted MPRAGE scan acquired on a Siemens MAGNETOM 3T Tim Trio MRI scanner (Invicro, London, UK). Molecular imaging included dynamic 90-min ^11^C-UCB-J PET scans (mean injected dose: 232.1 ± 47.7 MBq) and ^11^C-DASB PET scans (mean injected dose: 228.5 ± 54.5 MBq), both acquired on a PET-CT Biograph TruePoint scanner (Invicro, London, UK). ^11^C-UCB-J PET scans included arterial blood sampling and metabolite analysis. Additionally, SPECT DATSCAN™ imaging was performed approximately 4 ± 0.5 h post-injection of ^123^I-FP-CIT (mean injected dose: 186.9 ± 11.0 MBq) using a SPECT-CT scanner at King's College Hospital (London, UK).

SPECT DATSCAN™ data were not acquired for five HC subjects at baseline due to study dropout due to COVID-19. Of these, four completed both ^11^C-UCB-J PET and ^11^C-DASB PET scans at follow-up, but SPECT DATSCAN™ was not performed. An additional HC dropped out of the study before follow-up was complete, resulting in the absence of both ^11^C-UCB-J PET and SPECT DATSCAN™ data for this participant. Experimental variables for all molecular imaging acquisitions are summarised in Table 3, Table 4.

**Table 3:** Baseline molecular imaging experimental variables.

Variable mean (SD)	Parkinson's			Healthy Controls	*p*	
	All n = 33	iPD n = 27	LRRK2-PD n = 6	n = 25	HCvsAll	HCvsiPD
**^11^C-UCB-J**						
Dose, MBq	222.0 (47.8)	220.0 (50.8)	231.2 (33.0)	245.5 (45.1)	0.033∗	0.049∗
Injected mass, mg	3.2 (1.6)	3.4 (1.8)	2.4 (0.6)	3.5 (2.2)	0.567	0.826
Specific activity, GBq/mmol	27.0 (10.8)	25.8 (10.9)	32.5 (8.9)	28.1 (11.2)	0.783	0.516
Total motion, mm	15.3 (6.8)	14.8 (6.3)	17.9 (9.2)	12.4 (6.6)	0.083	0.136
Max interframe motion, mm	2.4 (1.7)	2.0 (1.1)	4.1 (2.8)	1.9 (2.3)	0.111	0.252
Scan Start Time	11:08:57	11:16:00	10:37:14	11:21:57	0.783	0.540
**^11^C-DASB**						
Dose, MBq	229.0 (55.2)	231.8 (53.6)	216.7 (65.7)	227.9 (54.8)	0.931	0.735
Injected mass, mg	1.4 (0.7)	1.5 (0.7)	1.1 (0.7)	2.0 (1.7)	0.405	0.654
Specific activity, GBq/mmol	52.0 (16.9)	49.4 (15.9)	63.5 (17.9)	46.2 (21.4)	0.289	0.615
Total motion, mm	19.3 (10.2)	19.0 (9.3)	20.5 (14.6)	13.8 (5.5)	0.024∗	0.027∗
Max interframe motion, mm	2.8 (1.9)	2.7 (1.5)	3.4 (3.3)	2.1 (0.9)	0.236	0.260
Scan start time	15:07:36	15:05:24	15:17:30	15:10:32	0.354	0.175
**DAT SPECT**				**n = 20**		
Dose, MBq	184.5 (9.7)	183.1 (8.7)	191.2 (12.1)	190.9 (12.1)	0.033∗	0.009∗
Scan start time	14:23:13	14:30:49	13:49:00	14:23:39	0.862	0.914

∗ Significant difference *p* < 0.05.

**Table 4:** Follow-up molecular imaging experimental variables.

Variable mean (SD)	Parkinson's	Healthy Controls	*p*
	n = 20		HCvsPD
**^11^C-UCB-J**		**n = 21**	
Dose, MBq	191.15 (61.5)	229.1 (61.4)	0.045∗
Injected mass, mg	3.5 (1.4)	3.5 (1.1)	0.744
Specific activity, GBq/mmol	19.4 (7.3)	23.2 (9.5)	0.220
Total motion, mm	16.3 (6.1)	12.2 (4.4)	0.011∗
Max interframe motion, mm	2.1 (1.5)	1.9 (1.5)	0.335
Time since baseline (days)	641 (250)	617 (199)	0.639
**^11^C-DASB**		**n = 22**	
Dose, MBq	204.4 (64.6)	235.8 (50.2)	0.096
Injected mass, mg	2.3 (1.9)	2.3 (1.8)	0.504
Specific activity, GBq/mmol	36.3 (16.4)	37.9 (13.4)	0.743
Total motion, mm	20.9 (7.4)	13.6 (5.7)	<0.001∗
Max interframe motion, mm	3.0 (1.8)	2.0 (1.5)	0.019∗
Time since baseline (days)	638 (242)	620 (209)	0.706
**DAT SPECT**		**n = 17**	
Dose, MBq	181.4 (11.5)	189.2 (9.5)	0.060
Time since baseline (days)	658 (246)	624 (227)	0.557
Scan start time	15:02:48	14:17:07	0.060

∗ Significant difference *p* < 0.05.

All the clinical and imaging assessments were performed in the OFF state, defined by protocol as after 12 h of withdrawal for immediate release and 24 h for controlled release dopaminergic supplementation. To allow this, the assessments were performed on different days, within a maximum of 2 months between them.

### 2.4 Image processing and quantification

Region-based morphometry (RBM) analysis was performed on the 3D T1-weighted MPRAGE images using the CAT12 toolbox (Gaser et al., 2024) in the Statistical Parametric Mapping (SPM12) software (Friston et al., 2006) implemented in MATLAB® (R2018b; The Mathworks, Natick, MA, USA). The processing included de-noising, partial volume estimation and normalization to MNI space using DARTEL (Ashburner, 2007). All outputs were visually inspected and the CAT12 processing report (including assessment of resolution, noise, and bias) was reviewed for each subject for quality assurance purposes. The output normalized and modulated partial volume grey matter maps were used to calculate regional grey matter volume by multiplying the mean partial volume of each region by the region volume. Regions of interest (ROIs) for RBM, ^11^C-UCB-J, and ^11^C-DASB analyses included occipital lobe, insular cortex, temporal lobe, frontal lobe, parietal lobe, thalamus, posterior cingulate, anterior cingulate, SN, caudate, and putamen defined based on the CIC v2.0 neuroanatomical atlas (Tziortzi et al., 2011).

Quantification of ^11^C-UCB-J using a one tissue compartmental model (1TCM) with arterial input provided a good fit and is the robust modelling method of choice to determine the total volume of distribution (V_T_) as the outcome measure (Mansur et al., 2020). Structural MRI and ^11^C-UCB-J PET image data were processed using an established analysis pipeline utilizing MIAKAT™ software v4.3.24, (Gunn et al., 2016) implemented in MATLAB® (R2018b; The MathWorks, Natick, MA, USA). MIAKAT™ uses a combination of SPM and FSL functions to accomplish brain extraction, segmentation, transformation of a neuroanatomical atlas to the PET images for ROI definition, image co-registration, motion correction, generation of a metabolite corrected plasma input function and kinetic modelling. All subjects were also processed using a simplified reference tissue model (SRTM) with centrum semiovale as a reference region and the binding potential (BP_ND_) as the main parameter of interest (Rossano et al., 2020). Grey matter ROIs were defined based on the CIC v2.0 neuroanatomical atlas and individual subject grey matter segmentation. All raw images, brain extracted images, segmentations, registration and motion correction steps, blood activity curves, plasma input function, data fits from kinetic modelling and ROI masks in the PET space were visually inspected for quality control purposes. Collection or quantification of blood data failed for four subjects at baseline (2 PD, 2 HC) and three subjects at follow-up (1 PD, 2 HC) and were therefore excluded from the 1TCM and only included in the SRTM analysis. All other subjects passed quality control checks.

The same MIAKAT™ software was also employed for pre-processing and analysis of ^11^C-DASB PET data. The SRTM was utilized for ^11^C-DASB PET quantification, using cerebellum grey matter (excluding vermis) as the reference region (Nørgaard et al., 2019). BP_ND_ was calculated as the main parameter of interest to represent serotonin transporter density in grey matter ROIs as defined based on the CIC v2.0 neuroanatomical atlas and individual subject grey matter segmentation images. All raw images, brain extracted images, segmentations, registration and motion correction steps and ROI masks in the PET space were visually inspected for quality control purposes. All subjects passed quality control checks.

**Fig. 1:**
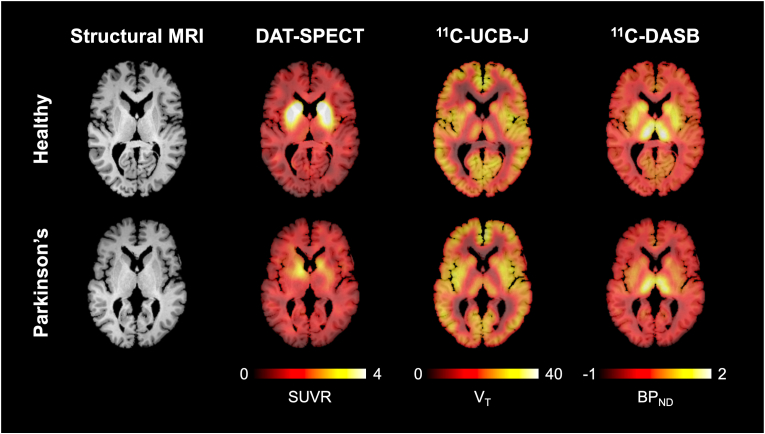
Representative imaging examples from a healthy control (55-year-old male) and an idiopathic Parkinson's disease patient (56-year-old male). From left to right, axial slices of brain extracted structural 3D T1-weighted MPRAGE magnetic resonance imaging (MRI), ^123^I-FP-CIT dopamine transporter (DAT) single-photon emission computed tomography (SPECT) standardised uptake value ratio (SUVR), ^11^C-UCB-J positron emission tomography (PET) total volume of distribution (V_T_), and ^11^C-DASB PET binding potential (BP_ND_) parametric maps. Molecular imaging parametric maps are overlaid on the corresponding structural MRI.

SPECT ^123^I-FP-CIT image volumes were spatially normalized to an ^123^I-FP-CIT template. All images were visually inspected for quality assurance and were included in the final analysis. Binding parameters were extracted using a standardised method provided by BRASS™ (Hermes Medical Solutions) embedded in the SPECT/CT scanner. The eight most prominent axial slices containing the striatum were summed and a standardised volume of interest (VOI) template was then applied to this image. VOI analyses were performed separately on the caudate and putamen, with results also combined to represent the striatum, using the occipital cortex region as the reference tissue (Vogt et al., 2011). Representative structural MRI as well as DAT SPECT SUVR, ^11^C-DASB BP_ND_, and ^11^C-UCB-J V_T_ parametric maps for one healthy control (55-year-old male) and one iPD subject (56-year-old male) are provided in Fig. 1.

### 2.5 Statistical analysis

All statistical analyses were conducted using SPSS (version 31.0.0.0; IBM, Armonk, NY). The Shapiro-Wilk test was employed to assess the normality of dependent variables. Group differences in experimental variables, body mass index (BMI), and clinical scores, including MDS-UPDRS, MDS-UPDRS III (motor symptom subscale), BDI, SCOPA-AUT, and MoCA, were evaluated using the Mann-Whitney *U* test, while age and years of education were assessed with the independent samples *t*-test. Difference in sex distribution between groups was evaluated using a chi-square test. Baseline group comparisons were performed between HC and all PD patients as well as between HC and iPD alone. A significance threshold of p = 0.05 was set for all analyses. As this was an exploratory analysis, p-values were reported without correction for multiple comparisons.

#### 2.5.1 Baseline cross-sectional

Analysis of covariance (ANCOVA) was conducted to examine differences in grey matter volume, ^11^C-UCB-J, ^11^C-DASB, and DAT SPECT measures between subject groups at baseline, with age and sex included as covariates. For ^11^C-UCB-J and DAT SPECT analyses, injected dose was assessed as an additional covariate, while total motion was assessed as a covariate for ^11^C-DASB group comparisons. These covariates were selected based on observed group differences in injected dose and estimated total motion among the experimental variables. To address potential partial volume effects, regional grey matter volume was also assessed as a covariate in ^11^C-UCB-J and ^11^C-DASB group comparisons.

Spearman's nonparametric rank correlation was used to examine relationships between molecular imaging metrics and clinical measures (MDS-UPDRS, MDS-UPDRS III, SCOPA-AUT, PDSS, ESS, RDBSQ, MCAS, PFS-16, KPPS, UPSIT, BDI, AES, MoCA, SDMT, and LNSI) within the PD group at baseline, with age and sex included as covariates. Due to the limited variability in clinical scores among healthy controls, correlations with molecular imaging metrics were not assessed in this group, as meaningful associations could not be reliably detected.

#### 2.5.2 Longitudinal

Group differences in time since baseline assessments were assessed using the Mann-Whitney *U* test. Linear mixed-effects models were employed to assess longitudinal changes in BMI and molecular imaging measures over time. Baseline age was included as a continuous covariate to adjust for age-related differences at the start of the study, while time (in days since baseline) was modelled as a continuous variable to evaluate longitudinal changes. Group (HC vs. PD) and sex were included as fixed factors, and participant ID was modelled as a random effect to account for repeated measurements within individuals. An interaction term (i.e., time × group) was included to test whether rates of change differed between groups.

Separate linear mixed-effects models were also applied to assess longitudinal changes in continuous clinical measures within the PD group, with time (in days since baseline) modelled as a continuous variable. Baseline age was incorporated as a continuous covariate, while sex was included as a fixed factor. These clinical measures included MDS-UPDRS, MDS-UPDRS III, SCOPA-AUT, PDSS, ESS, RDBSQ, MCAS, PFS-16, KPPS, UPSIT, BDI, AES, MoCA, SDMT, and LNSI. For assessing change in H&Y scores, generalized estimating equations (GEE) with an ordinal cumulative logit link function was utilized. Participant ID was included as a random effect in the mixed models and as a subject variable in the GEE to account for repeated measures within individuals.

## 3 Results

### 3.1 Demographic, clinical, and experimental group differences

No significant differences in age, sex distribution, BMI, or years of education were observed between HC and PD groups at either baseline or follow-up. PD patients consistently demonstrated significantly worse MDS-UPDRS total scores (baseline: U = 825, p < 0.001; follow-up: U = 675, p < 0.001), MDS-UPDRS III (baseline: U = 825, p < 0.001; follow-up: U = 675, p < 0.001), BDI (baseline: U = 695, p < 0.001; follow-up: U = 350.5, p < 0.001), and SCOPA-AUT (baseline: U = 657, p < 0.001; follow-up: U = 336, p = 0.003). While MoCA scores were significantly lower in PD compared to HC at baseline (U = 281, p = 0.036), this difference was not maintained when excluding the LRRK2-PD group and not maintained at follow-up. These group comparison results in demographic and clinical variables are summarised in Table 1, Table 2

Injected doses of ^11^C-UCB-J (U = 277, p = 0.033) and ^123^I-FP-CIT (U = 214, p = 0.033) were significantly higher in HC compared to PD at baseline. At follow-up, injected dose of ^11^C-UCB-J was significantly higher in HC (U = 133, p = 0.045). Total motion during ^11^C-DASB acquisition was significantly greater in PD compared to HC at baseline (U = 556, p = 0.024). At follow-up, total motion during ^11^C-UCB-J (U = 308, p = 0.011) and ^11^C-DASB acquisitions (U = 362, p < 0.001), as well as maximum interframe motion for ^11^C-DASB (U = 313, p = 0.019), were significantly greater in PD compared to HC. No other significant differences in clinical or experimental variables were identified. These group comparison results in experimental variables are summarised in Table 2, Table 3 for baseline and follow-up, respectively.

### 3.2 Baseline cross-sectional

#### 3.2.1 Structural imaging RBM

No significant grey matter volume differences between PD and HC were detected across the occipital lobe, insular cortex, temporal lobe, frontal lobe, parietal lobe, thalamus, posterior cingulate, anterior cingulate, SN, caudate, and putamen at baseline. These results were maintained when excluding the LRRK2-PD group.

#### 3.2.2 Dopamine transporter imaging

**Fig. 2:**
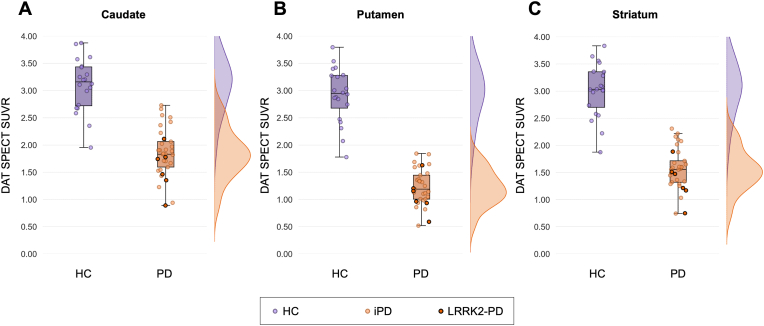
Boxplots comparing DAT SPECT standardised uptake value ratio (SUVR) values between healthy controls (HC) and Parkinson's disease (PD) groups across (A) Caudate (p < 0.001 HCvsAll; p < 0.001 HCvsiPD), (B) Putamen (p < 0.001 HCvsAll; p < 0.001 HCvsiPD), and (C) Striatum (p < 0.001 HCvsAll; p < 0.001 HCvsiPD) at baseline. Individual data points are displayed, with LRRK2-PD cases outlined in black within the PD group, which includes both idiopathic PD (iPD) and LRRK2-PD. Density plots illustrate the distribution of SUVR values for each region across each group.

Significantly lower DAT uptake represented by SUVR was observed in PD compared to HC across caudate (F(1,49) = 124.226, p < 0.001, $\eta_{\rho }^{2}$ = 0.717), putamen (F(1,49) = 271.020, p < 0.001, $\eta_{\rho }^{2}$ = 0.847), and striatum (F(1,49) = 190.139, p < 0.001, $\eta_{\rho }^{2}$ = 0.795) (Fig. 2). These results were maintained when excluding the LRRK2-PD subjects and were consistent when also correcting for injected dose. No Scans Without Evidence of Dopaminergic Deficit (SWEDDs) were observed in this study population.

UPSIT scores were positively correlated with DAT SUVR in the putamen (r(26) = 0.391, p = 0.040) and striatum (r(26) = 0.405, p = 0.032), while SDMT scores positively correlated with SUVR in the caudate (r(29) = 0.448, p = 0.011), putamen (r(29) = 0.420, p = 0.019), and striatum (r(29) = 0.452, p = 0.011). AES also positively correlated with SUVRs in striatum (r(28) = 0.366, p = 0.046). MDS-UPDRS III and total MDS-UPDRS scores were observed to be negatively associated with SUVR in caudate (UPDRS III: r(29) = -0.369, p = 0.041; total UPDRS: r(29) = -0.439, p = 0.014), putamen (UPDRS III: r(29) = -0.371, p = 0.040; total UPDRS: r(29) = -0.369, p = 0.041), and striatum (UPDRS III: r(29) = -0.384, p = 0.033; total UPDRS: r(29) = -0.426, p = 0.017). However, the only association retained after excluding the LRRK2-PD group was between caudate SUVR and SDMT (r(23) = 0.414, p = 0.040).

#### 3.2.3 Serotonin transporter imaging

**Fig. 3:**
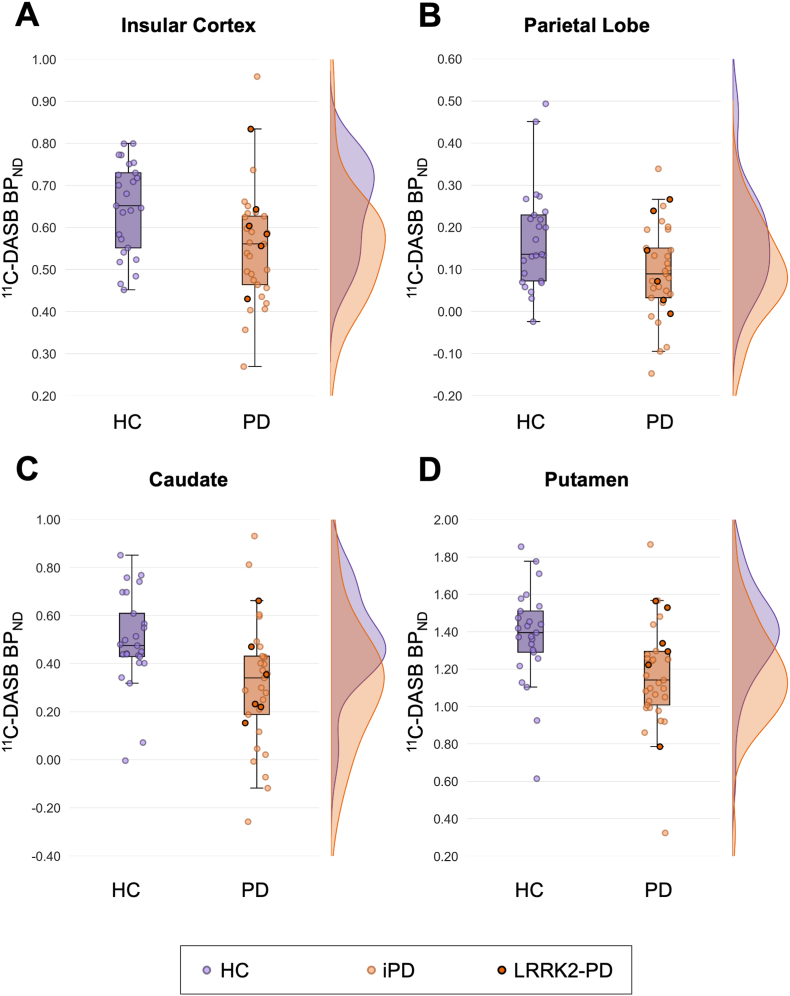
Boxplots comparing ^11^C-DASB BP_ND_ values between healthy controls (HC) and Parkinson's disease (PD) groups across four brain regions at baseline: (A) Insular cortex (p = 0.003 HCvsAll; p = 0.001 HCvsiPD), (B) Parietal lobe (p = 0.009 HCvsAll; p = 0.009 HCvsiPD), (C) Caudate (p < 0.001 HCvsAll; p = 0.001 HCvsiPD), and (D) Putamen (p = 0.002 HCvsAll; p < 0.001 HCvsiPD). Individual data points are displayed, with LRRK2-PD cases outlined in black within the PD group, which includes both idiopathic PD (iPD) and LRRK2-PD. Density plots illustrate the distribution of BP_ND_ values for each region across each group.

Significantly lower ^11^C-DASB BP_ND_ was observed in PD compared to HC in insular cortex (HCvsAll: F(1,54) = 9.832, p = 0.003, $\eta_{\rho }^{2}$ = 0.154; HCvsiPD: F(1,48) = 11.595, p = 0.001, $\eta_{\rho }^{2}$ = 0.195), parietal lobe (HCvsAll: F(1,54) = 7.264, p = 0.009, $\eta_{\rho }^{2}$ = 0.119; HCvsiPD: F(1,48) = 7.358, p = 0.009, $\eta_{\rho }^{2}$ = 0.133), caudate (HCvsAll: F(1,54) = 12.957, p < 0.001, $\eta_{\rho }^{2}$ = 0.193; HCvsiPD: F(1,48) = 11.372, p = 0.001, $\eta_{\rho }^{2}$ = 0.192), and putamen (HCvsAll: F(1,54) = 10.751, p = 0.002, $\eta_{\rho }^{2}$ = 0.166; HCvsiPD: F(1,48) = 12.386, p < 0.001, $\eta_{\rho }^{2}$ = 0.205) (Fig. 3). These results remained consistent when also controlling for total motion and regional grey matter volume, except for in parietal lobe, which no longer reached statistical significance (HCvsAll: F(1,52) = 3.711, p = 0.060, $\eta_{\rho }^{2}$ = 0.067; HCvsiPD: F(1,46) = 3.996, p = 0.052, $\eta_{\rho }^{2}$ = 0.080). Significantly lower ^11^C-DASB BP_ND_ in the occipital (F(1,48) = 5.221, p = 0.027, $\eta_{\rho }^{2}$ = 0.098) and frontal (F(1,48) = 4.258, p = 0.044, $\eta_{\rho }^{2}$ = 0.081) lobes was observed when comparing the iPD group alone to HC, which remained consistent when also correcting for total motion and regional grey matter volume.

In the combined PD group, significant positive associations were observed between anterior cingulate ^11^C-DASB BP_ND_ and both LNSI (r(29) = 0.420, p = 0.019) and MDS-UPDRS III (r(29) = 0.437, p = 0.014). Putamen BP_ND_ also showed a positive correlation with ESS (r(29) = 0.449, p = 0.011). When excluding the LRRK2-PD group, the association between anterior cingulate BP_ND_ and LNSI remained significant (r(23) = 0.431, p = 0.032), but other significant correlations were no longer observed. A new significant positive association emerged between SN BP_ND_ and UPSIT scores (r(20) = 0.484, p = 0.022).

#### 3.2.4 Synaptic density imaging

No significant differences in ^11^C-UCB-J uptake, represented by V_T_ or BP_ND_ were observed between PD and HC across occipital lobe, insular cortex, temporal lobe, frontal lobe, parietal lobe, thalamus, posterior cingulate, anterior cingulate, SN, caudate, and putamen (Supplemental Figs. 1 and 2). These results were maintained when excluding the LRRK2-PD subjects and were consistent when also correcting for injected dose and regional grey matter volume.

When considering ^11^C-UCB-J uptake as V_T_ in the combined PD group, significant positive associations were observed between RBDSQ scores and V_T_ across all analysed regions except the SN. These included the occipital lobe (r(27) = 0.459, p = 0.012), insular cortex (r(27) = 0.503, p = 0.005), temporal lobe (r(27) = 0.518, p = 0.004), frontal lobe (r(27) = 0.458, p = 0.013), parietal lobe (r(27) = 0.392, p = 0.036), thalamus (r(27) = 0.514, p = 0.004), posterior cingulate (r(27) = 0.504, p = 0.005), anterior cingulate (r(27) = 0.501, p = 0.006), caudate (r(27) = 0.423, p = 0.022), and putamen (r(27) = 0.504, p = 0.005). Additionally, SCOPA-AUT scores were positively associated with V_T_ in the SN (r(27) = 0.378, p = 0.043) and caudate (r(27) = 0.397, p = 0.033). When excluding the LRRK2-PD group, a significant positive association between RBDSQ and SN V_T_ emerged (r(22) = 0.418, p = 0.042), while associations with SCOPA-AUT were no longer significant. Correlations were then also observed between caudate V_T_ with PFS-16 (r(22) = 0.411, p = 0.046) and KPPS (r(22) = 0.488, p = 0.015).

For ^11^C-UCB-J BP_ND_ in the combined PD group, significant positive correlations were observed between SCOPA-AUT and BP_ND_ in the temporal lobe (r(29) = 0.359, p = 0.047) and SN (r(29) = 0.628, p < 0.001). SN BP_ND_ also showed a positive correlation with KPPS (r(29) = 0.416, p = 0.020). MoCA scores positively correlated with BP_ND_ across multiple regions: occipital lobe (r(29) = 0.453, p = 0.011), insular cortex (r(29) = 0.577, p < 0.001), temporal lobe (r(29) = 0.441, p = 0.013), thalamus (r(29) = 0.398, p = 0.027), posterior cingulate (r(29) = 0.467, p = 0.008), anterior cingulate (r(29) = 0.495, p = 0.005), caudate (r(29) = 0.370, p = 0.040), and putamen (r(29) = 0.476, p = 0.007). Conversely, MDS-UPDRS III scores negatively correlated with BP_ND_ in the parietal lobe (r(29) = -0.398, p = 0.026), posterior cingulate (r(29) = -0.396, p = 0.027), and putamen (r(29) = -0.424, p = 0.017).

When excluding the LRRK2-PD group, SCOPA-AUT also correlated positively with BP_ND_ in the frontal lobe (r(23) = 0.444, p = 0.026), anterior cingulate (r(23) = 0.469, p = 0.018), and putamen (r(23) = 0.450, p = 0.024). PFS-16 also correlated positively with SN BP_ND_ (r(23) = 0.406, p = 0.044). However, the only positive correlation retained with MoCA was with insular cortex BP_ND_ (r(23) = 0.419, p = 0.037). Original correlations with MDS-UPDRS III were no longer significant, but a new negative correlation emerged between MDS-UPDRS III and thalamus BP_ND_ (r(23) = -0.409, p = 0.042). New negative correlations also emerged between SDMT and occipital lobe (r(23) = -0.549, p = 0.004), temporal lobe (r(23) = -0.471, p = 0.017), frontal lobe (r(23) = -0.546, p = 0.005), parietal lobe (r(23) = -0.586, p = 0.002), thalamus (r(23) = -0.400, p = 0.048), posterior cingulate (r(23) = -0.548, p = 0.005), anterior cingulate (r(23) = -0.576, p = 0.016), and caudate (r(23) = -0.401, p = 0.047) BP_ND_.

A qualitative summary of the correlation results between molecular imaging metrics and clinical scores in the PD group is provided in Supplemental Table 1.

### 3.3 Longitudinal

PD patients showed a significant increase in levodopa equivalent daily dose (LEDD) over time (β = 0.234, F = 12.418, p = 0.002). Clinical measures in the PD group worsened from baseline to follow-up, with significant increases in SCOPA-AUT (β = 0.003, F = 9.797, p = 0.006), MDS-UPDRS III (β = 0.008, F = 7.452, p = 0.013), and total MDS-UPDRS (β = 0.012, F = 6.117, p = 0.023), indicating a progression of autonomic dysfunction, motor impairment, and overall disability (Table 2). In contrast, MoCA scores significantly improved over time (β = 0.002, F = 11.012, p = 0.004) (Supplemental Fig. 3). No other significant time effects were observed for H&Y scores or for any other clinical variables.

A significant effect of time was observed for regional grey matter volume across both HC and PD groups, with reductions across all regions (p < 0.001) except for the thalamus and SN. However, no significant group effects or group × time interactions were found for regional grey matter volume (Supplemental Fig. 4).

Significant group differences were observed in DAT uptake, with lower values in PD compared to HC across both time points in the caudate (SUVR: β = 1.356, F = 98.232, p < 0.001), putamen (SUVR: β = 1.785, F = 206.539, p < 0.001), and striatum (SUVR: β = 1.556, F = 145.502, p < 0.001) (Supplemental Fig. 5). Similarly, ^11^C-DASB BP_ND_ was significantly lower in PD compared to HC in the insular cortex (β = 0.098, F = 9.508, p = 0.003), parietal lobe (β = 0.079, F = 6.822, p = 0.011), caudate (β = 0.201, F = 12.786, p < 0.001), and putamen (β = 0.224, F = 10.716, p = 0.002) (Supplemental Fig. 6).

In contrast, no significant group differences were observed for ^11^C-UCB-J BP_ND_ or V_T_ across all regions and time points (Supplemental Figs. 7 and 8). No significant main effect of time or group × time interaction was observed for any molecular imaging measure, indicating that although some group differences were present across both time points, there were no detectable longitudinal changes over the follow-up period.

The means and standard deviations of all baseline imaging metrics (including grey matter volumes, DAT SPECT SUVRs, ^11^C-DASB BP_ND_, ^11^C-UCB-J BP_ND_, and ^11^C-UCB-J V_T_) are detailed in Supplemental Tables 2–6. Test statistics from statistical analyses are provided in Supplemental Tables 7–20.

## 4 Discussion

This study is the first to simultaneously assess dopaminergic, serotonergic, and synaptic density molecular imaging biomarkers within the same cohort, comparing patients with mild-to-moderate PD to matched HC in both cross-sectional and longitudinal analyses. Consistent with prior studies, DAT uptake was significantly lower in PD compared to HC, reflecting the well-documented decline in pre-synaptic dopaminergic integrity in PD. However, no significant longitudinal change in DAT binding was observed, despite clinical progression in motor impairment, autonomic dysfunction, and overall disability. These findings suggest a relatively stable dopaminergic trajectory in PD at this disease stage and align with previous findings (Vogt et al., 2011) indicating that DAT imaging may have limited prognostic utility over short-to-intermediate follow-up periods. This limitation is likely due to the previously reported floor effect, whereby the greatest annualized decline in DAT binding occurs within the first year after diagnosis (Simuni et al., 2018). More broadly, these results highlight the complex and heterogeneous nature of PD progression, where clinical decline does not necessarily align with measurable changes in dopaminergic markers.

At baseline, DAT uptake was negatively correlated with motor impairment and overall disability, indicating greater reductions in pre-synaptic dopaminergic integrity in individuals with more severe symptoms. While this relationship has been previously reported (Vogt et al., 2011; Honhar et al., 2024; Lorio et al., 2019; Kerstens et al., 2023), other investigations have found weak or inconsistent associations with motor scores (Delva et al., 2020; Fazio et al., 2018; Jakobson Mo et al., 2018). We also found reduced DAT uptake linked to slower processing speeds and worse cognitive performance (assessed with SDMT), consistent with a prior report (Andersson et al., 2021). This suggests that dopaminergic integrity may also contribute to cognitive performance in PD, particularly in domains such as attention and processing speed.

Taken together, these findings reinforce the utility of DAT imaging as a valuable tool for assessing dopaminergic degeneration and its relation to both motor and non-motor symptoms in PD. However, the absence of significant longitudinal changes in both imaging and clinical measures limits our ability to assess the relationship between dopaminergic decline and symptom progression over time. Capturing disease progression at earlier stages - before substantial dopaminergic loss - and/or extending follow-up durations may be crucial for clarifying these relationships and enhancing the use of molecular imaging biomarkers in tracking PD progression.

The significant reductions in ^11^C-DASB binding in PD relative to controls, particularly in the insular cortex, parietal lobe, caudate, and putamen were accompanied by moderate to large effect sizes, supporting the biological relevance of these differences despite the exploratory design. The most consistent finding was a significant reduction in the putamen, aligning with established evidence of serotonergic dysfunction (Pagano et al., 2017; Guttman et al., 2007) and its unique spatial pattern (Fu et al., 2018) in PD. These results reinforce previous reports of widespread serotonergic changes across various PD stages (Pagano et al., 2017; Guttman et al., 2007; Fu et al., 2018), although no longitudinal changes were observed.

Importantly, our findings are also consistent with earlier neuropathological studies demonstrating marked serotonergic disturbances in PD, including substantial interindividual variability in serotonergic neuron loss and terminal innervation (Kish et al., 2008). These observations suggest that serotonergic degeneration does not follow a uniform trajectory across individuals. A previous study suggested that SERT remains unaffected in early-stage PD (2.1 ± 0.4 months post-diagnosis) in non-depressed individuals, even when DAT levels were already reduced (Strecker et al., 2011). Our findings indicate that serotonergic changes occur within the first 5 years of PD, as our cohort had an average disease duration of 5.2 ± 3.6 years at baseline.

While only weak correlations were observed, ^11^C-DASB binding was positively associated with motor impairment, daytime sleepiness, olfactory function, and working memory capacity. These findings are partially consistent with previous research. A positive relationship between SERT binding and motor impairment in cervical dystonia has previously been reported (Smit et al., 2018), and SERT activity has been linked to dyskinesia in PD (Lee et al., 2015). A prior study found positive associations between SERT and fatigue (Pavese et al., 2010). However, in this study we observed a relationship with daytime sleepiness (measured with ESS) rather than overall fatigue (measured with PFS-16) and the correlation between SERT and daytime sleepiness was negative. Although the current study showed a weak positive association between olfactory function and ^11^C-DASB, a previous study found no significant relationship in the absence of dementia (Bohnen and Müller, 2013), suggesting SERT may play a minimal role in olfactory deficits. We observed that working memory and general cognitive performance (assessed with LNSI) were positively associated with ^11^C-DASB binding, particularly in the anterior cingulate cortex - a region modulated by serotonergic neurotransmission and involved in working memory (Xu et al., 2024). These findings align with a previous report linking SERT levels to cognitive processes (Štrac et al., 2016). Although higher SERT levels have been correlated with worse depressive symptoms in PD patients with depression (Boileau et al., 2008), our cohort generally had minimal depressive symptoms (BDI = 8.4 ± 6.4), limiting our ability to assess this relationship.

Of note, the amount of radiolabelled ^11^C-DASB administered for the SERT PET scans in this study was lower than previously reported (Guttman et al., 2007; Fu et al., 2018; Strecker et al., 2011). Importantly, these lower doses achieved reliable and quantifiable data, demonstrating that lower doses are viable for clinical studies. This finding supports the ethical justification for reducing the radioactive dose of ^11^C-DASB in future clinical research with this PET tracer.

No statistically significant differences in ^11^C-UCB-J binding metrics (V_T_ or BP_ND_) representing synapse density were observed between HC and PD, nor were any longitudinal changes detected in this study. Although moderate effect sizes were observed for group differences in the SN and caudate ($\eta_{\rho }^{2}$ ≈ 0.060, Supplemental Tables 10 and 11), these did not reach statistical significance and should therefore be interpreted cautiously. Given that ^11^C-UCB-J is a marker of synaptic density, these findings suggest that this tracer may lack sensitivity in detecting PD-related synaptic changes at mild-to-moderate disease stages, characterised by relatively low H&Y stages (1-3), mild motor symptoms, minimal fluctuations, and absence of cognitive impairment. This contrasts with prior studies that reported synaptic density loss at similar disease stages, particularly in the SN (Holmes et al., 2024; Delva et al., 2020; Matuskey et al., 2020; Martin et al., 2023; Wilson et al., 2020) highlighting the potential limitations of ^11^C-UCB-J PET imaging in PD or the need for larger, longer-term studies to detect subtle progressive changes.

Despite the absence of significant group differences, baseline clinical correlations suggest that synaptic integrity remains relevant to disease severity. Lower ^11^C-UCB-J binding was associated with greater motor impairment, suggesting a role for synaptic loss in motor dysfunction. Greater synaptic density measures were linked to increased autonomic dysfunction, fatigue, and pain, suggesting possible compensatory mechanisms or non-motor synaptic alterations. Additionally, higher ^11^C-UCB-J binding was associated with better overall cognitive performance, aligning with previous studies in PD (Andersen et al., 2021) and Alzheimer's disease (Mecca et al., 2022). Significant positive correlations with symptoms of REM sleep behaviour disorder and synaptic density were observed across all brain regions analysed, which may indicate that ^11^C-UCB-J is sensitive to synaptic density changes associated with REM sleep disturbances. Given that selective synaptic pruning and strengthening predominantly occurs during REM sleep (Li et al., 2017), this relationship warrants further investigation.

A significant reduction in grey matter volume was observed across both groups over time, generally consistent with normal aging (Neufeld et al., 2022). Grey matter volume was not observed to be a significant driver of molecular imaging results, suggesting that the observed differences in DAT, SERT, and synaptic density markers were independent of structural atrophy. No other significant changes in imaging markers were observed over the follow-up period. While PD progressed clinically, molecular imaging markers remained insensitive to these changes, raising questions about their utility in tracking disease progression over this timeframe and disease stage.

The observed cognitive improvement in our PD cohort may help explain why some expected trajectories were not detected. This finding contrasts with previous longitudinal work reporting progressive annual declines in MoCA performance among patients with middle-age onset PD. Several factors may account for these differences (Kim et al., 2020). Potential effects of COVID-19 should also be considered, as some studies suggest that social isolation accelerates cognitive decline in PD (Huang et al., 2023). However, some participants may have had greater social interactions during the pandemic due to family members or carers spending more time at home, which may have counteracted these negative effects. Additionally, despite the time gap between assessments, practice effects from repeated cognitive testing could have influenced cognitive results (Lei et al., 2022). These findings emphasize the need for future studies to consider follow-up duration, cognitive performance stability, and potential external factors when assessing longitudinal changes in PD.

## 5 Limitations

Partial volume correction (PVC) was not applied during pre-processing of ^11^C-UCB-J or ^11^C-DASB data. This decision was made due to the absence of a standardised method for PVC and the potential for PVC to introduce variability (Erlandsson et al., 2012), reducing statistical power. To address potential partial volume effects, we confirmed the absence of significant grey matter volume differences between PD and HC groups. Additionally, we validated the robustness of our findings by including regional grey matter volume as a covariate in cross-sectional group comparisons and confirmed that the results remained stable. This represents a conservative approach, as PET signal reduction are often colinear with tissue loss, and adjusting for volume may therefore underestimate true molecular alterations.

We note that absolute BP_ND_ values may differ across studies depending on preprocessing choices such as PVC. Therefore, the reference values reported here should be interpreted within the context of the applied methodology, with particular utility for within-study and similarly processed datasets.

In addition, a small number of ^11^C-DASB scans exhibited unexpectedly low BP_ND_ values. Similar outliers have been reported in previous ^11^C-DASB PET studies in Parkinson's disease, and in the present study these scans constituted only a small proportion of the total dataset. Importantly, all key statistical findings remained stable when these scans were excluded, indicating that they did not drive the reported results.

Although some statistically significant associations between clinical and molecular imaging measures were identified, the exploratory nature of the analysis and the number of statistical tests conducted make interpretation challenging. Multiple comparison correction was not applied due to the study's exploratory design. However, this approach allows identification of potential trends that can inform future research, and all summary test statistics are provided in the Supplement to support this aim. In particular, some region-specific trends between ^11^C-DASB and ^11^C-UCB-J uptake and clinical measures were observed. These findings are presented as hypothesis-generating, with caution against over-interpretation. Additionally, the absence of significant associations between some clinical measures and ^11^C-DASB or ^11^C-UCB-J uptake may reflect the relatively mild symptom severity and limited variability within the patient cohort. Future studies with larger cohorts and pre-specified regional hypotheses will be needed to clarify the pathophysiological relevance of these region-specific associations.

The relatively small sample size, particularly for the LRRK2-PD group, is another limitation that impacts the generalizability of our findings. Visual inspection of the baseline plots suggests that LRRK2-PD participants are generally evenly distributed within the iPD group and do not exhibit distinct patterns in DAT, ^11^C-DASB, or ^11^C-UCB-J binding, although larger sample sizes will be needed to confirm this. This is consistent with previous imaging reports suggesting that LRRK2-PD shares broadly similar dopaminergic and serotonergic alterations with idiopathic PD, albeit with variable severity across studies (Nandhagopal et al., 2008; Wile et al., 2017). Recruitment challenges due to the COVID-19 pandemic constrained the study's power to detect effects across iPD, LRRK2-PD, and HC groups. Future studies with larger cohorts are needed to further explore and understand the differences between LRRK2-driven and idiopathic PD.

An additional limitation of this study is the heterogeneity in disease duration at baseline among PD participants, which is likely to contribute to inter-individual variability in longitudinal PET trajectories. Furthermore, visual inspection of longitudinal plots of clinical measures (Supplementary Fig. 3) suggests that attrition was more prevalent among participants with greater baseline clinical severity, particularly those with lower MoCA and higher SCOPA-AUT scores. This pattern of non-random attrition may bias longitudinal estimates toward individuals with milder disease and potentially attenuate observed rates of change over time. These factors should therefore be considered when interpreting the longitudinal findings and underscore the need for future studies with larger cohorts and improved strategies to retain participants with greater clinical burden.

Additionally, the average follow-up period in our cohort was approximately 20 months, which may not have been sufficient to detect longitudinal changes in markers such as ^11^C-UCB-J as previously suggested (Delva et al., 2022). The relevance of synaptic density to cognition in PD, as indicated by both prior (Andersen et al., 2021) and current findings, may also be a contributing factor, as our cohort did not exhibit worsening cognitive performance over the follow-up period. Longer follow-up durations may be necessary to capture subtler alterations in synaptic density over time in mild-to-moderate PD. However, extended follow-ups pose additional challenges, as increased disease burden can lead to greater motion artifacts and patient discomfort, potentially compromising data quality. Balancing follow-up duration with data quality and participant well-being will be an important consideration for future studies.

Finally, heterogeneity in follow-up timing was an unavoidable limitation. COVID-19-related disruptions led to delays in imaging, clinical, and cognitive assessments for some participants, resulting in variable follow-up intervals. However, the longitudinal statistical models explicitly accounted for time since baseline for each assessment, and there was limited variability in the patient cohort. These factors suggest that variability in follow-up timing is unlikely to have significantly influenced the findings.

## 6 Summary/conclusion

The multi-modal approach adopted in this study provides valuable insights, consistently revealing significantly reduced striatal DAT uptake and widespread serotonergic alterations in PD compared to HC. In contrast, no significant differences in synaptic density, as assessed by ^11^C-UCB-J PET, were observed, highlighting the potential limitations of this marker in detecting synaptic loss at mild-to-moderate disease stages. Relationships between imaging metrics and clinical measures revealed associations between synaptic density and motor, cognitive, and non-motor symptoms, offering new perspectives on how neurotransmitter and synaptic alterations relate to PD's diverse clinical phenotype. These findings underscore the importance of integrating molecular imaging with clinical data to advance our understanding of PD pathophysiology and inform biomarker development.

## CRediT authorship contribution statement

**Julia J. Schubert:** Writing – original draft, Visualization, Software, Methodology, Formal analysis, Data curation. **Silvia Rota:** Writing – review & editing, Visualization, Validation, Resources, Project administration, Investigation, Data curation, Conceptualization. **Lucia Batzu:** Writing – review & editing, Visualization, Validation, Resources, Project administration, Investigation, Data curation, Conceptualization. **Pavlos Zinzalias:** Writing – review & editing, Visualization, Resources, Project administration, Investigation, Data curation. **Nazneen Barwick:** Writing – review & editing, Conceptualization. **Sarah Lee:** Writing – review & editing, Project administration. **Mattia Veronese:** Writing – review & editing, Project administration, Methodology, Investigation, Data curation. **Alastair D. Reith:** Writing – review & editing, Supervision, Project administration, Funding acquisition, Conceptualization. **Christine A. Parker:** Writing – review & editing, Visualization, Validation, Project administration, Methodology, Conceptualization. **Steve C.R. Williams:** Writing – review & editing, Supervision, Project administration, Funding acquisition, Conceptualization.

## Declaration of competing interest

The authors declare the following financial interests/personal relationships which may be considered as potential competing interests:Authors JJS, SR, LB, PZ, and MV declare no financial or non-financial competing interests. Authors CAP, ADR, and NB were or are employees of GlaxoSmithKline (GSK), a global healthcare company that may conceivably benefit financially through this publication. SL is a company director of Amallis Consulting LTD and is a paid consultant for GSK, but declares no non-financial competing interests. SCRW served as the principal investigator on this project, which was supported by funding from GSK to King's College London.

## Data Availability

The data that support the findings of this study (both original and processed) have been digitally notarised into blockchain and are available from the corresponding author upon reasonable request.
