# Supplementary material for: Molecular neuroimaging of Parkinson's disease: association of motor and non-motor symptoms with synaptic density, dopaminergic and serotonergic systems
Source: Neuroimage Rep. 2026 Mar 31;6(2):100337. doi: 10.1016/j.ynirp.2026.100337 (PMC13081182; doi:10.1016/j.ynirp.2026.100337)
Supplement: Multimedia component 1 [file mmc1.docx]

**Molecular neuroimaging of Parkinson’s disease: association of motor and non-motor symptoms with Synaptic Density, Dopaminergic and Serotonergic systems**

Julia J. Schubert et al.

**Supplementary Material**

**Supplemental Figure 1.** Boxplots comparing ^11^C-UCB-J V_T_ values between healthy controls (HC) and Parkinson’s disease (PD) groups across four brain regions at baseline: (A) Insular cortex (p=0.672 HCvsAll; p=0.455 HCvsiPD), (B) Parietal lobe (p=0.528 HCvsAll; p=0.695 HCvsiPD), (C) Caudate (p=0.071 HCvsAll; p=0.145 HCvsiPD), and (D) Putamen (p=0.581 HCvsAll; p=0.852 HCvsiPD). Individual data points are displayed, with LRRK2-PD cases outlined in black within the PD group, which includes both idiopathic PD (iPD) and LRRK2-PD. Density plots illustrate the distribution of V_T_ values for each region across each group.

**Supplemental Figure 2.** Boxplots comparing ^11^C-UCB-J BP_ND_ values between healthy controls (HC) and Parkinson’s disease (PD) groups across four brain regions at baseline: (A) Insular cortex (p=0.935 HCvsAll; p=0.578 HCvsiPD), (B) Parietal lobe (p=0.776 HCvsAll; p=0.868 HCvsiPD), (C) Caudate (p=0.148 HCvsAll; p=0.258 HCvsiPD), and (D) Putamen (p=0.662 HCvsAll; p=942 HCvsiPD). Individual data points are displayed, with LRRK2-PD cases outlined in black within the PD group, which includes both idiopathic PD (iPD) and LRRK2-PD. Density plots illustrate the distribution of BP_ND_ values for each region across each group.

**Supplemental Figure 3.** Spaghetti plots illustrating individual trajectories of clinical measures in the Parkinson’s disease (PD) group from baseline to follow-up, including (A) levodopa equivalent daily dose (LEDD), (B) Scales for Outcomes in Parkinson’s Disease-Autonomic (SCOPA-AUT), (C) Movement Disorder Society-Unified PD Rating Scale (MDS-UPDRS) III, (D) total MDS-UPDRS, and (E) Montreal Cognitive Assessment (MoCA) scores. Each point represents an individual measurement; lines connect repeated measures within the same participant. LRRK2-PD cases are outlined in black within the PD group, which includes both idiopathic PD (iPD) and LRRK2-PD. All baseline datapoints are shown although some participants do not have follow-up measurements.

**Supplemental Figure 4.** Spaghetti plots showing individual trajectories of regional grey matter from baseline to follow-up in healthy controls (HC) and Parkinson’s disease (PD) groups across four regions: (A) Insular Cortex, (B) Parietal Lobe, (C) Caudate, and (D) Putamen. Each point represents an individual measurement; lines connect repeated measures within the same participant. Within the PD group, LRRK2-PD cases are outlined in black, with the remaining PD cases representing idiopathic PD (iPD). All baseline datapoints are shown, although some participants do not have follow-up measurements.

**Supplemental Figure 5.** Spaghetti plots showing individual trajectories of DAT SPECT standardised uptake value ratio (SUVR) values from baseline to follow-up in healthy controls (HC) and Parkinson’s disease (PD) groups across (A) Caudate, (B) Putamen, and (C) Striatum. Each point represents an individual measurement; lines connect repeated measures within the same participant. Within the PD group, LRRK2-PD cases are outlined in black, with the remaining PD cases representing idiopathic PD (iPD). All baseline datapoints are shown, although some participants do not have follow-up measurements.

**Supplemental Figure 6.** Spaghetti plots showing individual trajectories of ¹¹C-DASB BP_ND_ from baseline to follow-up in healthy controls (HC) and Parkinson’s disease (PD) groups across four regions: (A) Insular Cortex, (B) Parietal Lobe, (C) Caudate, and (D) Putamen. Each point represents an individual measurement; lines connect repeated measures within the same participant. Within the PD group, LRRK2-PD cases are outlined in black, with the remaining PD cases representing idiopathic PD (iPD). All baseline datapoints are shown, although some participants do not have follow-up measurements.

**Supplemental Figure 7.** Spaghetti plots showing individual trajectories of ¹¹C-UCB-J BP_ND_ from baseline to follow-up in healthy controls (HC) and Parkinson’s disease (PD) groups across four regions: (A) Insular Cortex, (B) Parietal Lobe, (C) Caudate, and (D) Putamen. Each point represents an individual measurement; lines connect repeated measures within the same participant. Within the PD group, LRRK2-PD cases are outlined in black, with the remaining PD cases representing idiopathic PD (iPD). All baseline datapoints are shown, although some participants do not have follow-up measurements.

**Supplemental Figure 8.** Spaghetti plots showing individual trajectories of ¹¹C-UCB-J V_T_ from baseline to follow-up in healthy controls (HC) and Parkinson’s disease (PD) groups across four regions: (A) Insular Cortex, (B) Parietal Lobe, (C) Caudate, and (D) Putamen. Each point represents an individual measurement; lines connect repeated measures within the same participant. Within the PD group, LRRK2-PD cases are outlined in black, with the remaining PD cases representing idiopathic PD (iPD). All baseline datapoints are shown, although some participants do not have follow-up measurements.

**Supplemental Table 1.** Qualitative summary of significant correlations between clinical assessments and molecular imaging measures in Parkinson’s disease (PD) at baseline. The table presents the direction of significant relationships (positive or negative) identified across **any** brain region investigated. For each clinical assessment and imaging measure pair, when a significant correlation was identified, the direction of the association was consistent across all implicated brain regions. Results are shown for standardized uptake value ratios (SUVR) for dopamine transporter (DAT) SPECT, binding potential (BP_ND_​) for ¹¹C-DASB, and both volume of distribution (V_T_​) and BP_ND_​ for ¹¹C-UCB-J. Idiopathic PD (iPD) and LRRK2-PD cases were included within the combined PD group, with group-specific findings noted where applicable. **Please see main text for a full quantitative description of these significant correlations, including correlation coefficients and p-values for all regions, molecular imaging measures, and clinical assessments. Test statistics are also provided in Supplemental Tables 12-15 below.**

| **Clinical Assessment** | **Interpretation** | **Imaging Measure** | | | |
| --- | --- | --- | --- | --- | --- |
|  |  | **DAT SPECT** | **^11^C-DASB** | **^11^C-UCB-J** | |
|  |  | **SUVR** | **BP_ND_** | **V_T_** | **BP_ND_** |
| **Total**  **MDS-UPDRS** | Higher score indicates greater severity of Parkinson's disease symptoms and functional disability | Negative  (combined PD group only) | - | - | - |
| **MDS-UPDRS III** | Higher score indicates greater severity of motor impairment | Negative  (combined PD group only) | Positive  (combined PD group only) | - | Negative |
| **SCOPA-AUT** | Higher score indicates more autonomic dysfunction | - | - | Positive  (combined PD group only) | Positive |
| **PDSS** | Lower score indicates more significant sleep problems | - | - | - | - |
| **ESS** | Higher score indicates greater daytime sleepiness | - | Positive  (combined PD group only) | - | - |
| **RBDSQ** | Higher score indicates greater levels of REM sleep behaviour disorder symptoms | - | - | Positive | - |
| **MCAS** | Higher score indicates greater degree of bowel function problems | - | - | - | - |
| **PFS-16** | Higher score indicates greater fatigue | - | - | Positive  (iPD only) | Positive  (iPD only) |
| **KPPS** | Higher score indicates greater overall burden of pain | - | - | Positive  (iPD only) | Positive |
| **UPSIT** | Lower score indicates worse olfactory function | Positive  (combined PD group only) | Positive  (iPD only) | - | - |
| **BDI** | Higher score indicates greater depression severity | - | - | - | - |
| **AES** | Higher score indicates more apathy | Positive  (combined PD group only) | - | - | - |
| **MoCA** | Lower score indicates greater cognitive impairment | - | - | - | Positive |
| **SDMT** | Lower score indicates slower processing speeds and general cognitive decline | Positive | - | - | Negative  (iPD only) |
| **LNSI** | Lower score indicates lower working memory capacity and general cognitive decline | - | Positive | - | - |

**Supplemental Table 2.** Baseline mean (± standard deviation (SD)) grey matter volumes (mm³) for regions of interest analysed using Region-Based Morphometry. Values are reported separately for healthy controls, all Parkinson’s disease (PD) participants (All), as well as separately for idiopathic PD (iPD) and LRRK2-PD groups.

| **Volume mm^3^**  **(mean ± SD)** | **Healthy**  **Controls** | **Parkinson’s** | | |
| --- | --- | --- | --- | --- |
|  | **n = 25** | **All**  **n = 33** | **iPD**  **n = 27** | **LRRK2-PD**  **n = 6** |
| **Occipital lobe** | 77,719.06 ± 10,994.01 | 74,679.53 ± 10,486.83 | 77,118.67 ± 8,341.71 | 63,703.38 ± 12,856.75 |
| **Insular cortex** | 11,340.21 ± 1,363.30 | 11,195.83 ± 1,521.11 | 11,500.68 ± 1,216.80 | 9,824.00 ± 2,085.39 |
| **Temporal lobe** | 122,033.70 ± 16,471.40 | 117,127.23 ± 14,562.21 | 120,258.52 ± 11,599.52 | 103,036.42 ± 19,139.17 |
| **Frontal lobe** | 167,526.39 ± 25,395.55 | 164,002.14 ± 19,285.47 | 168,191.76 ± 16,538.38 | 145,148.86 ± 20,898.12 |
| **Parietal lobe** | 103,764.06 ± 14,790.52 | 98,472.84 ± 11,348.79 | 100,893.56 ± 9,287.46 | 87,579.59 ± 14,202.58 |
| **Thalamus** | 10,590.81 ± 1,420.87 | 10,334.82 ± 1,181.46 | 10,512.70 ± 1,076.37 | 9,534.36 ± 1,403.21 |
| **Posterior cingulate** | 10,318.18 ± 1,571.39 | 9,944.51 ± 1,268.11 | 10,174.30 ± 1,094.24 | 8,910.49 ± 1,580.32 |
| **Anterior cingulate** | 28,221.02 ± 4,579.90 | 28,085.62 ± 3,487.75 | 28,852.71 ± 2,937.58 | 24,633.71 ± 3,937.40 |
| **Substantia nigra** | 16.67 ± 10.27 | 19.90 ± 18.93 | 20.90 ± 20.43 | 15.37 ± 9.69 |
| **Caudate** | 4,818.90 ± 618.06 | 4,555.98 ± 678.56 | 4,703.44 ± 587.89 | 3,892.41 ± 709.80 |
| **Putamen** | 7,043.58 ± 824.69 | 6,737.00 ± 930.07 | 6,871.92 ± 802.18 | 6,129.85 ± 1,284.30 |

**Supplemental Table 3.** Baseline mean (± standard deviation (SD)) dopamine transporter (DAT) SPECT standardised uptake value ratios (SUVRs) for regions of interest. Values are reported separately for healthy controls, all Parkinson’s disease (PD) participants (All), as well as separately for idiopathic PD (iPD) and LRRK2-PD groups.

| **SUVR**  **(mean ± SD)** | **Healthy**  **Controls** | **Parkinson’s** | | |
| --- | --- | --- | --- | --- |
|  | **n = 20** | **All**  **n = 33** | **iPD**  **n = 27** | **LRRK2-PD**  **n = 6** |
| **Caudate** | 3.10 ± 0.49 | 1.85 ± 0.44 | 1.92 ± 0.43 | 1.56 ± 0.42 |
| **Putamen** | 2.92 ± 0.51 | 1.23 ± 0.33 | 1.26 ± 0.32 | 1.08 ± 0.35 |
| **Striatum** | 3.01 ± 0.49 | 1.56 ± 0.38 | 1.61 ± 0.37 | 1.34 ± 0.38 |

**Supplemental Table 4.** Baseline mean (± standard deviation (SD)) ^11^C-DASB binding potentials (BP_ND_) for regions of interest. Values are reported separately for healthy controls, all Parkinson’s disease (PD) participants (All), as well as separately for idiopathic PD (iPD) and LRRK2-PD groups.

| **BP_ND_**  **(mean ± SD)** | **Healthy**  **Controls** | **Parkinson’s** | | |
| --- | --- | --- | --- | --- |
|  | **n = 25** | **All**  **n = 33** | **iPD**  **n = 27** | **LRRK2-PD**  **n = 6** |
| **Occipital lobe** | 0.19 ± 0.10 | 0.15 ± 0.10 | 0.13 ± 0.07 | 0.22 ± 0.18 |
| **Insular cortex** | 0.65 ± 0.11 | 0.56 ± 0.13 | 0.54 ± 0.14 | 0.61 ± 0.13 |
| **Temporal lobe** | 0.35 ± 0.07 | 0.31 ± 0.12 | 0.31 ± 0.13 | 0.34 ± 0.06 |
| **Frontal lobe** | 0.21 ± 0.14 | 0.15 ± 0.11 | 0.14 ± 0.11 | 0.21 ± 0.10 |
| **Parietal lobe** | 0.17 ± 0.12 | 0.10 ± 0.11 | 0.09 ± 0.11 | 0.12 ± 0.11 |
| **Thalamus** | 1.15 ± 0.37 | 1.11 ± 0.56 | 1.15 ± 0.61 | 0.94 ± 0.21 |
| **Posterior cingulate** | 0.41 ± 0.16 | 0.35 ± 0.12 | 0.35 ± 0.13 | 0.37 ± 0.09 |
| **Anterior cingulate** | 0.51 ± 0.11 | 0.46 ± 0.18 | 0.46 ± 0.19 | 0.46 ± 0.09 |
| **Substantia nigra** | 2.40 ± 0.55 | 2.24 ± 0.79 | 2.11 ± 0.77 | 2.80 ± 0.64 |
| **Caudate** | 0.50 ± 0.20 | 0.32 ± 0.26 | 0.31 ± 0.27 | 0.35 ± 0.19 |
| **Putamen** | 1.38 ± 0.26 | 1.17 ± 0.28 | 1.14 ± 0.28 | 1.29 ± 0.28 |

**Supplemental Table 5.** Baseline mean (± standard deviation (SD)) ^11^C-UCB-J volume of distribution (V_T_) for regions of interest. Values are reported separately for healthy controls, all Parkinson’s disease (PD) participants (All), as well as separately for idiopathic PD (iPD) and LRRK2-PD groups.

| **V_T_**  **(mean ± SD)** | **Healthy**  **Controls** | **Parkinson’s** | | |
| --- | --- | --- | --- | --- |
|  | **n = 23** | **All**  **n = 31** | **iPD**  **n = 26** | **LRRK2-PD**  **n = 5** |
| **Occipital lobe** | 18.32 ± 2.48 | 18.12 ± 1.92 | 18.26 ± 1.79 | 17.36 ± 2.58 |
| **Insular cortex** | 21.39 ± 3.01 | 21.59 ± 2.27 | 21.85 ± 2.20 | 20.21 ± 2.41 |
| **Temporal lobe** | 19.81 ± 2.75 | 19.67 ± 2.06 | 19.85 ± 1.98 | 18.75 ± 2.46 |
| **Frontal lobe** | 17.86 ± 2.36 | 18.01 ± 2.01 | 18.15 ± 1.96 | 17.28 ± 2.31 |
| **Parietal lobe** | 18.10 ± 2.43 | 17.76 ± 2.01 | 17.87 ± 1.88 | 17.22 ± 2.80 |
| **Thalamus** | 13.68 ± 2.30 | 13.44 ± 1.51 | 13.56 ± 1.53 | 12.85 ± 1.40 |
| **Posterior cingulate** | 20.48 ± 2.90 | 20.35 ± 2.12 | 20.47 ± 1.97 | 19.71 ± 2.98 |
| **Anterior cingulate** | 20.91 ± 2.84 | 21.16 ± 2.37 | 21.35 ± 2.23 | 20.18 ± 3.08 |
| **Substantia nigra** | 9.37 ± 1.04 | 8.84 ± 1.06 | 8.87 ± 1.14 | 8.68 ± 0.54 |
| **Caudate** | 14.40 ± 2.63 | 13.52 ± 1.97 | 13.61 ± 1.92 | 13.03 ± 2.39 |
| **Putamen** | 22.90 ± 2.99 | 22.39 ± 2.50 | 22.70 ± 2.45 | 20.78 ± 2.30 |

**Supplemental Table 6.** Baseline mean (± standard deviation (SD)) ^11^C-UCB-J binding potentials (BP_ND_) for regions of interest. Values are reported separately for healthy controls, all Parkinson’s disease (PD) participants (All), as well as separately for idiopathic PD (iPD) and LRRK2-PD groups.

| **BP_ND_**  **(mean ± SD)** | **Healthy**  **Controls** | **Parkinson’s** | | |
| --- | --- | --- | --- | --- |
|  | **n = 25** | **All**  **n = 33** | **iPD**  **n = 27** | **LRRK2-PD**  **n = 6** |
| **Occipital lobe** | 2.15 ± 0.50 | 2.12 ± 0.51 | 2.19 ± 0.45 | 1.79 ± 0.69 |
| **Insular cortex** | 2.73 ± 0.75 | 2.73 ± 0.68 | 2.84 ± 0.57 | 2.25 ± 0.97 |
| **Temporal lobe** | 2.44 ± 0.66 | 2.41 ± 0.61 | 2.50 ± 0.52 | 2.02 ± 0.86 |
| **Frontal lobe** | 2.10 ± 0.57 | 2.10 ± 0.59 | 2.18 ± 0.50 | 1.76 ± 0.85 |
| **Parietal lobe** | 2.14 ± 0.55 | 2.10 ± 0.57 | 2.18 ± 0.51 | 1.77 ± 0.78 |
| **Thalamus** | 1.39 ± 0.48 | 1.37 ± 0.43 | 1.44 ± 0.36 | 1.07 ± 0.60 |
| **Posterior cingulate** | 2.55 ± 0.59 | 2.56 ± 0.61 | 2.64 ± 0.51 | 2.20 ± 0.92 |
| **Anterior cingulate** | 2.61 ± 0.65 | 2.64 ± 0.73 | 2.74 ± 0.61 | 2.22 ± 1.10 |
| **Substantia nigra** | 0.58 ± 0.27 | 0.47 ± 0.21 | 0.50 ± 0.20 | 0.37 ± 0.23 |
| **Caudate** | 1.48 ± 0.54 | 1.29 ± 0.62 | 1.35 ± 0.49 | 1.03 ± 1.04 |
| **Putamen** | 2.87 ± 0.62 | 2.79 ± 0.75 | 2.90 ± 0.62 | 2.29 ± 1.12 |

**Supplemental Table 7.** F-values and p-values from baseline ANCOVA analyses comparing grey matter volumes between healthy controls (n = 25, HC) and Parkinson’s disease (PD) groups. Comparisons were performed for HC vs. all PD (n = 33, including idiopathic PD (iPD) and LRRK2-PD; HCvsAll) and HC vs. iPD only (n = 27; HCvsiPD). Due to limited sample sizes, statistical comparisons across all three groups independently (i.e., HC, iPD, and LRRK2-PD) were not feasible. Covariates included age and sex.

|  |  | **HCvsAll** | | | **HCvsiPD** | | |
| --- | --- | --- | --- | --- | --- | --- | --- |
|  | | **F** | ***p*** | $\boldsymbol{\eta}_{\boldsymbol{\rho}}^{\boldsymbol{2}}$ | **F** | ***p*** | $\boldsymbol{\eta}_{\boldsymbol{\rho}}^{\boldsymbol{2}}$ |
| **Occipital lobe** | | 0.883 | 0.352 | 0.016 | 0.049 | 0.825 | 0.001 |
| **Insular cortex** | | 0.002 | 0.966 | 0.000 | 0.444 | 0.508 | 0.009 |
| **Temporal lobe** | | 1.118 | 0.295 | 0.020 | 0.210 | 0.648 | 0.004 |
| **Frontal lobe** | | 0.102 | 0.751 | 0.002 | 0.049 | 0.825 | 0.001 |
| **Parietal lobe** | | 2.550 | 0.116 | 0.045 | 1.039 | 0.313 | 0.021 |
| **Thalamus** | | 0.302 | 0.585 | 0.006 | 0.035 | 0.852 | 0.001 |
| **Posterior cingulate** | | 0.739 | 0.394 | 0.013 | 0.163 | 0.688 | 0.003 |
| **Anterior cingulate** | | 0.038 | 0.846 | 0.001 | 0.547 | 0.463 | 0.011 |
| **Substantia nigra** | | 1.169 | 0.284 | 0.021 | 1.342 | 0.252 | 0.027 |
| **Caudate** | | 1.899 | 0.174 | 0.034 | 0.495 | 0.485 | 0.010 |
| **Putamen** | | 1.434 | 0.236 | 0.026 | 0.645 | 0.426 | 0.013 |

**Supplemental Table 8.** F-values and p-values from baseline ANCOVA analyses comparing dopamine transporter (DAT) SPECT standardised uptake value ratios (SUVRs) between healthy controls (n = 20, HC) and Parkinson’s disease (PD) groups. Comparisons were performed for HC vs. all PD (n = 33, including idiopathic PD (iPD) and LRRK2-PD; HCvsAll) and HC vs. iPD only (n = 27; HCvsiPD). Due to limited sample sizes, statistical comparisons across all three groups independently (i.e., HC, iPD, and LRRK2-PD) were not feasible. Covariates (covar) always included age and sex, with additional models incorporating injected dose. Statistically significant results (p<0.05) are highlighted with bold font.

|  |  | **HCvsAll** | | | | | | **HCvsiPD** | | | | | |
| --- | --- | --- | --- | --- | --- | --- | --- | --- | --- | --- | --- | --- | --- |
|  | **Covar** | **Age and Sex** | | | **+Injected Dose** | | | **Age and Sex** | | | **+Injected Dose** | | |
|  | | **F** | ***p*** | $\boldsymbol{\eta}_{\boldsymbol{\rho}}^{\boldsymbol{2}}$ | **F** | ***p*** | $\boldsymbol{\eta}_{\boldsymbol{\rho}}^{\boldsymbol{2}}$ | **F** | ***p*** | $\boldsymbol{\eta}_{\boldsymbol{\rho}}^{\boldsymbol{2}}$ | **F** | ***p*** | $\boldsymbol{\eta}_{\boldsymbol{\rho}}^{\boldsymbol{2}}$ |
| **Caudate** | | **124.226** | **<0.001** | **0.717** | **124.679** | **<0.001** | **0.722** | **128.281** | **<0.001** | **0.749** | **108.528** | **<0.001** | **0.721** |
| **Putamen** | | **271.020** | **<0.001** | **0.847** | **237.715** | **<0.001** | **0.832** | **262.075** | **<0.001** | **0.859** | **209.838** | **<0.001** | **0.833** |
| **Striatum** | | **190.139** | **<0.001** | **0.795** | **176.996** | **<0.001** | **0.787** | **193.306** | **<0.001** | **0.818** | **157.291** | **<0.001** | **0.789** |

**Supplemental Table 9.** F-values and p-values from baseline ANCOVA analyses comparing ^11^C-DASB BP_ND_ between healthy controls (n = 25, HC) and Parkinson’s disease (PD) groups. Comparisons were performed for HC vs. all PD (n = 33, including idiopathic PD (iPD) and LRRK2-PD; HCvsAll) and HC vs. iPD only (n = 27; HCvsiPD). Due to limited sample sizes, statistical comparisons across all three groups independently (i.e., HC, iPD, and LRRK2-PD) were not feasible. Covariates (covar) included age and sex, with additional models incorporating total motion and regional grey matter (GM) volume. Statistically significant results (p<0.05) are highlighted with bold font.

|  |  | **HCvsAll** | | | | | | **HCvsiPD** | | | | | |
| --- | --- | --- | --- | --- | --- | --- | --- | --- | --- | --- | --- | --- | --- |
|  | **Covar** | **Age and Sex** | | | **+Total Motion**  **GM Volume** | | | **Age and Sex** | | | **+Total motion**  **GM volume** | | |
|  | | **F** | ***p*** | $\boldsymbol{\eta}_{\boldsymbol{\rho}}^{\boldsymbol{2}}$ | **F** | ***p*** | $\boldsymbol{\eta}_{\boldsymbol{\rho}}^{\boldsymbol{2}}$ | **F** | ***p*** | $\boldsymbol{\eta}_{\boldsymbol{\rho}}^{\boldsymbol{2}}$ | **F** | ***p*** | $\boldsymbol{\eta}_{\boldsymbol{\rho}}^{\boldsymbol{2}}$ |
| **Occipital lobe** | | 2.283 | 0.137 | 0.041 | 1.274 | 0.264 | 0.024 | **5.221** | **0.027** | **0.098** | **4.637** | **0.037** | **0.092** |
| **Insular cortex** | | **9.832** | **0.003** | **0.154** | **8.773** | **0.005** | **0.144** | **11.595** | **0.001** | **0.195** | **12.201** | **0.001** | **0.210** |
| **Temporal lobe** | | 2.206 | 0.143 | 0.039 | 2.052 | 0.158 | 0.038 | 2.568 | 0.116 | 0.051 | 2.718 | 0.106 | 0.056 |
| **Frontal lobe** | | 3.254 | 0.077 | 0.057 | 3.014 | 0.088 | 0.055 | **4.258** | **0.044** | **0.081** | **4.322** | **0.043** | **0.086** |
| **Parietal lobe** | | **7.264** | **0.009** | **0.119** | 3.711 | 0.060 | 0.067 | **7.358** | **0.009** | **0.133** | 3.996 | 0.052 | 0.080 |
| **Thalamus** | | 0.329 | 0.569 | 0.006 | 0.164 | 0.687 | 0.003 | 0.039 | 0.844 | 0.001 | 0.049 | 0.825 | 0.001 |
| **Posterior cingulate** | | 3.195 | 0.079 | 0.056 | 2.169 | 0.147 | 0.040 | 3.111 | 0.084 | 0.061 | 1.718 | 0.196 | 0.036 |
| **Anterior cingulate** | | 1.618 | 0.209 | 0.029 | 1.582 | 0.214 | 0.030 | 1.489 | 0.228 | 0.030 | 1.483 | 0.230 | 0.031 |
| **Substantia nigra** | | 0.860 | 0.358 | 0.016 | 1.395 | 0.243 | 0.026 | 2.653 | 0.110 | 0.052 | 3.963 | 0.052 | 0.079 |
| **Caudate** | | **12.957** | **<0.001** | **0.193** | **10.621** | **0.002** | **0.170** | **11.372** | **0.001** | **0.192** | **9.183** | **0.004** | **0.166** |
| **Putamen** | | **10.751** | **0.002** | **0.166** | **7.652** | **0.008** | **0.128** | **12.386** | **<0.001** | **0.205** | **10.096** | **0.003** | **0.180** |

**Supplemental Table 10.** F-values and p-values from baseline ANCOVA analyses comparing ^11^C-UCB-J V_T_ between healthy controls (n = 23, HC) and Parkinson’s disease (PD) groups. Comparisons were performed for HC vs. all PD (n = 31, including idiopathic PD (iPD) and LRRK2-PD; HCvsAll) and HC vs. iPD only (n = 26; HCvsiPD). Due to limited sample sizes, statistical comparisons across all three groups independently (i.e., HC, iPD, and LRRK2-PD) were not feasible. Covariates (covar) included age and sex, with additional models incorporating injected dose and grey matter (GM) volume.

|  |  | **HCvsAll** | | | | | | **HCvsiPD** | | | | | |
| --- | --- | --- | --- | --- | --- | --- | --- | --- | --- | --- | --- | --- | --- |
|  | **Covar** | **Age and Sex** | | | **+Injected Dose**  **GM Volume** | | | **Age and Sex** | | | **+Injected Dose**  **GM volume** | | |
|  | | **F** | ***p*** | $\boldsymbol{\eta}_{\boldsymbol{\rho}}^{\boldsymbol{2}}$ | **F** | ***p*** | $\boldsymbol{\eta}_{\boldsymbol{\rho}}^{\boldsymbol{2}}$ | **F** | ***p*** | $\boldsymbol{\eta}_{\boldsymbol{\rho}}^{\boldsymbol{2}}$ | **F** | ***p*** | $\boldsymbol{\eta}_{\boldsymbol{\rho}}^{\boldsymbol{2}}$ |
| **Occipital lobe** | | 0.101 | 0.751 | 0.002 | 0.014 | 0.905 | 0.000 | 0.003 | 0.955 | 0.000 | 0.077 | 0.783 | 0.002 |
| **Insular cortex** | | 0.181 | 0.672 | 0.004 | 0.397 | 0.532 | 0.008 | 0.568 | 0.455 | 0.012 | 0.924 | 0.342 | 0.021 |
| **Temporal lobe** | | 0.004 | 0.952 | 0.000 | 0.068 | 0.796 | 0.001 | 0.028 | 0.868 | 0.001 | 0.167 | 0.685 | 0.004 |
| **Frontal lobe** | | 0.129 | 0.721 | 0.003 | 0.462 | 0.500 | 0.010 | 0.362 | 0.551 | 0.008 | 0.653 | 0.424 | 0.015 |
| **Parietal lobe** | | 0.404 | 0.528 | 0.008 | 0.005 | 0.943 | 0.000 | 0.156 | 0.695 | 0.003 | 0.016 | 0.900 | 0.000 |
| **Thalamus** | | 0.441 | 0.510 | 0.009 | 0.076 | 0.785 | 0.002 | 0.081 | 0.777 | 0.002 | 0.005 | 0.942 | 0.000 |
| **Posterior cingulate** | | 0.021 | 0.886 | 0.000 | 0.040 | 0.842 | 0.001 | 0.003 | 0.954 | 0.000 | 0.119 | 0.732 | 0.003 |
| **Anterior cingulate** | | 0.241 | 0.625 | 0.005 | 0.477 | 0.493 | 0.010 | 0.593 | 0.445 | 0.013 | 0.883 | 0.353 | 0.020 |
| **Substantia nigra** | | 3.165 | 0.081 | 0.060 | 3.447 | 0.068 | 0.068 | 2.473 | 0.123 | 0.052 | 2.781 | 0.103 | 0.061 |
| **Caudate** | | 2.401 | 0.071 | 0.064 | 1.994 | 0.164 | 0.040 | 2.200 | 0.145 | 0.047 | 1.333 | 0.255 | 0.030 |
| **Putamen** | | 0.308 | 0.581 | 0.006 | 0.374 | 0.544 | 0.008 | 0.035 | 0.852 | 0.001 | 0.045 | 0.834 | 0.001 |

**Supplemental Table 11.** F-values and p-values from baseline ANCOVA analyses comparing ^11^C-UCB-J BP_ND_ between healthy controls (n = 25, HC) and Parkinson’s disease (PD) groups. Comparisons were performed for HC vs. all PD (n = 33, including idiopathic PD (iPD) and LRRK2-PD; HCvsAll) and HC vs. iPD only (n = 27; HCvsiPD). Due to limited sample sizes, statistical comparisons across all three groups independently (i.e., HC, iPD, and LRRK2-PD) were not feasible. Covariates (covar) included age and sex, with additional models incorporating injected dose and grey matter (GM) volume.

|  |  | **HCvsAll** | | | | | | **HCvsiPD** | | | | | |
| --- | --- | --- | --- | --- | --- | --- | --- | --- | --- | --- | --- | --- | --- |
|  | **Covar** | **Age and Sex** | | | **+Injected Dose**  **GM Volume** | | | **Age and Sex** | | | **+Injected Dose**  **GM volume** | | |
|  | | **F** | ***p*** | $\boldsymbol{\eta}_{\boldsymbol{\rho}}^{\boldsymbol{2}}$ | **F** | ***p*** | $\boldsymbol{\eta}_{\boldsymbol{\rho}}^{\boldsymbol{2}}$ | **F** | ***p*** | $\boldsymbol{\eta}_{\boldsymbol{\rho}}^{\boldsymbol{2}}$ | **F** | ***p*** | $\boldsymbol{\eta}_{\boldsymbol{\rho}}^{\boldsymbol{2}}$ |
| **Occipital lobe** | | 0.053 | 0.819 | 0.001 | 0.894 | 0.349 | 0.017 | 0.052 | 0.821 | 0.001 | 1.211 | 0.277 | 0.026 |
| **Insular cortex** | | 0.007 | 0.935 | 0.000 | 0.098 | 0.756 | 0.002 | 0.313 | 0.578 | 0.006 | 0.247 | 0.622 | 0.005 |
| **Temporal lobe** | | 0.005 | 0.947 | 0.000 | 0.535 | 0.468 | 0.010 | 0.118 | 0.733 | 0.002 | 0.921 | 0.342 | 0.020 |
| **Frontal lobe** | | 0.021 | 0.886 | 0.000 | 0.294 | 0.590 | 0.006 | 0.308 | 0.582 | 0.006 | 0.512 | 0.478 | 0.011 |
| **Parietal lobe** | | 0.082 | 0.776 | 0.002 | 0.727 | 0.398 | 0.014 | 0.028 | 0.868 | 0.001 | 1.332 | 0.254 | 0.028 |
| **Thalamus** | | 0.054 | 0.817 | 0.001 | 0.470 | 0.496 | 0.009 | 0.100 | 0.754 | 0.002 | 1.296 | 0.261 | 0.027 |
| **Posterior cingulate** | | 0.030 | 0.864 | 0.001 | 1.151 | 0.288 | 0.022 | 0.334 | 0.566 | 0.007 | 1.855 | 0.176 | 0.039 |
| **Anterior cingulate** | | 0.099 | 0.754 | 0.002 | 0.210 | 0.648 | 0.004 | 0.573 | 0.453 | 0.012 | 0.391 | 0.535 | 0.008 |
| **Substantia nigra** | | 3.211 | 0.079 | 0.056 | 1.827 | 0.182 | 0.034 | 1.852 | 0.180 | 0.037 | 0.823 | 0.369 | 0.018 |
| **Caudate** | | 2.156 | 0.148 | 0.038 | 0.816 | 0.371 | 0.015 | 1.308 | 0.258 | 0.027 | 0.546 | 0.464 | 0.012 |
| **Putamen** | | 0.194 | 0.662 | 0.004 | 0.028 | 0.867 | 0.001 | 0.005 | 0.942 | 0.000 | 0.051 | 0.823 | 0.001 |

**Supplemental Table 12.** Spearman’s correlation coefficients (r) and p-values for associations between baseline clinical scores and dopamine transporter (DAT) SPECT standardised uptake value ratios (SUVRs) in the Parkinson’s disease (PD) group (n = 33), where idiopathic PD (n = 27) and LRRK2-PD (n = 6) cases were included. Note that University of Pennsylvania Smell Identification Test (UPSIT) scores were not collected for three participants, and Apathy Evaluation Scale (AES) score was not collected for one participant. Age and sex were included as covariates. Statistically significant results (p<0.05) are highlighted with bold font.

|  | **Total MDS-UPDRS** | | **MDS-UPDRS**  **III** | | **SCOPA-AUT** | | **PDSS** | | **ESS** | | **RBDSQ** | | **MCAS** | | **PFS-16** | | **KPPS** | | **UPSIT** | | **BDI** | | **AES** | | **MoCA** | | **SDMT** | | **LNSI** | |
| --- | --- | --- | --- | --- | --- | --- | --- | --- | --- | --- | --- | --- | --- | --- | --- | --- | --- | --- | --- | --- | --- | --- | --- | --- | --- | --- | --- | --- | --- | --- |
|  | **r** | ***p*** | **r** | ***p*** | **r** | ***p*** | **r** | ***p*** | **R** | ***p*** | **r** | ***p*** | **r** | ***p*** | **r** | ***p*** | **r** | ***p*** | **r** | ***p*** | **r** | ***p*** | **r** | ***p*** | **r** | ***p*** | **r** | ***p*** | **r** | ***p*** |
| **Caudate** | **-0.439** | **0.014** | **-0.369** | **0.041** | 0.004 | 0.983 | 0.196 | .0.292 | -0.090 | 0.629 | -0.084 | 0.653 | -0.274 | 0.135 | -0.228 | 0.218 | -0.343 | 0.059 | 0.369 | 0.053 | -0.186 | 0.317 | 0.327 | 0.078 | 0.194 | 0.296 | **0.448** | **0.011** | 0.108 | 0.563 |
| **Putamen** | **-0.369** | **0.041** | **-0.371** | **0.040** | 0.097 | 0.603 | 0.023 | 0.904 | 0.118 | 0.527 | 0.030 | 0.872 | -0.154 | 0.409 | -0.062 | 0.740 | -0.158 | 0.395 | **0.391** | **0.040** | -0.032 | 0.863 | 0.336 | 0.069 | 0.149 | 0.423 | **0.420** | **0.019** | 0.036 | 0.850 |
| **Striatum** | **-0.426** | **0.017** | **-0.384** | **0.033** | 0.046 | 0.808 | 0.121 | 0.516 | 0.016 | 0.931 | -0.014 | 0.940 | -0.217 | 0.242 | -0.155 | 0.404 | -0.243 | 0.188 | **0.405** | **0.032** | -0.148 | 0.428 | **0.366** | **0.046** | 0.176 | 0.344 | **0.452** | **0.011** | -0.073 | 0.698 |

**Supplemental Table 13.** Spearman’s correlation coefficients (r) and p-values for associations between baseline clinical scores and ^11^C-DASB BP_ND_ in the Parkinson’s disease (PD) group (n = 33), where idiopathic PD (n = 27) and LRRK2-PD (n = 6) cases were included. Note that University of Pennsylvania Smell Identification Test (UPSIT) scores were not collected for three participants, and Apathy Evaluation Scale (AES) score was not collected for one participant. Age and sex were included as covariates. Statistically significant results (p<0.05) are highlighted with bold font.

|  | **Total MDS-UPDRS** | | **MDS-UPDRS**  **III** | | **SCOPA-AUT** | | **PDSS** | | **ESS** | | **RBDSQ** | | **MCAS** | | **PFS-16** | | **KPPS** | | **UPSIT** | | **BDI** | | **AES** | | **MoCA** | | **SDMT** | | **LNSI** | |
| --- | --- | --- | --- | --- | --- | --- | --- | --- | --- | --- | --- | --- | --- | --- | --- | --- | --- | --- | --- | --- | --- | --- | --- | --- | --- | --- | --- | --- | --- | --- |
|  | **r** | ***p*** | **r** | ***p*** | **r** | ***p*** | **r** | ***p*** | **r** | ***p*** | **r** | ***p*** | **r** | ***p*** | **r** | ***p*** | **r** | ***p*** | **r** | ***p*** | **r** | ***p*** | **r** | ***p*** | **r** | ***p*** | **r** | ***p*** | **r** | ***p*** |
| **Occipital lobe** | 0.082 | 0.662 | 0.128 | 0.494 | 0.025 | 0.892 | 0.124 | 0.507 | -0.134 | 0.471 | -0.106 | 0.569 | 0.126 | 0.499 | -0.232 | 0.209 | -0.215 | 0.246 | -0.019 | 0.924 | -0.017 | 0.929 | 0.103 | 0.589 | -0.265 | 0.150 | -0.144 | 0.438 | 0.002 | 0.991 |
| **Insular cortex** | 0.248 | 0.179 | 0.332 | 0.068 | -0.090 | 0.630 | -0.083 | 0.656 | 0.178 | 0.337 | -0.280 | 0.127 | 0.090 | 0.631 | 0.004 | 0.985 | -0.057 | 0.761 | 0.051 | 0.797 | 0.068 | 0.717 | -0.005 | 0.979 | -0.170 | 0.361 | 0.029 | 0.879 | 0.080 | 0.667 |
| **Temporal lobe** | 0.303 | 0.098 | 0.333 | 0.067 | -0.016 | 0.933 | -0.219 | 0.236 | 0.175 | 0.345 | -0.142 | 0.445 | 0.174 | 0.349 | -0.032 | 0.863 | 0.133 | 0.477 | 0.109 | 0.581 | 0.083 | 0.655 | -0.073 | 0.703 | -0.280 | 0.127 | -0.092 | 0.622 | -0.041 | 0.827 |
| **Frontal lobe** | 0.182 | 0.328 | 0.229 | 0.215 | 0.094 | 0.615 | 0.169 | 0.364 | 0.049 | 0.794 | 0.045 | 0.809 | 0.348 | 0.055 | 0.030 | 0.873 | 0.076 | 0.684 | -0.263 | 0.176 | 0.072 | 0.699 | -0.143 | 0.451 | -0.086 | 0.645 | -0.127 | 0.497 | 0.162 | 0.385 |
| **Parietal lobe** | -0.078 | 0.678 | -0.045 | 0.808 | -0.045 | 0.810 | 0.278 | 0.129 | -0.195 | 0.293 | 0.011 | 0.953 | 0.225 | 0.224 | -0.169 | 0.363 | -0.172 | 0.355 | -0.195 | 0.319 | -0.077 | 0.682 | 0.156 | 0.411 | -0.030 | 0.872 | -0.072 | 0.700 | 0.219 | 0.237 |
| **Thalamus** | -0.196 | 0.290 | -0.131 | 0.484 | -0.009 | 0.963 | 0.204 | 0.272 | 0.059 | 0.752 | -0.154 | 0.408 | 0.064 | 0.734 | -0.025 | 0.893 | -0.063 | 0.738 | 0.021 | 0.915 | -0.110 | 0.554 | 0.344 | 0.063 | 0.184 | 0.321 | 0.272 | 0.139 | 0.360 | 0.094 |
| **Posterior cingulate** | 0.005 | 0.978 | 0.033 | 0.862 | 0.050 | 0.788 | 0.183 | 0.324 | -0.115 | 0.539 | 0.161 | 0.388 | 0.324 | 0.075 | -0.067 | 0.719 | -0.155 | 0.406 | -0.109 | 0.582 | 0.026 | 0.890 | 0.098 | 0.607 | -0.128 | 0.492 | -0.097 | 0.603 | 0.214 | 0.247 |
| **Anterior cingulate** | 0.297 | 0.105 | **0.437** | **0.014** | -0.091 | 0.628 | -0.073 | 0.697 | 0.162 | 0.383 | -0.025 | 0.892 | 0.075 | 0.689 | 0.120 | 0.520 | 0.074 | 0.691 | 0.063 | 0.748 | 0.087 | 0.641 | 0.037 | 0.848 | -0.144 | 0.439 | 0.029 | 0.875 | **0.420** | **0.019** |
| **Substantia nigra** | 0.219 | 0.237 | 0.102 | 0.585 | 0.351 | 0.053 | -0.183 | 0.324 | 0.166 | 0.373 | 0.162 | 0.383 | 0.270 | 0.142 | 0.342 | 0.060 | 0.248 | 0.179 | 0.368 | 0.054 | 0.129 | 0.489 | 0.027 | 0.888 | -0.188 | 0.310 | -0.184 | 0.322 | -0.121 | 0.517 |
| **Caudate** | 0.101 | 0.590 | 0.102 | 0.586 | -0.033 | 0.862 | -0.115 | 0.540 | 0.298 | 0.103 | -0.251 | 0.174 | -0.044 | 0.814 | 0.018 | 0.923 | 0.194 | 0.296 | -0.043 | 0.826 | 0.125 | 0.503 | -0.103 | 0.590 | 0.258 | 0.161 | 0.155 | 0.404 | 0.133 | 0.476 |
| **Putamen** | -0.083 | 0.659 | -0.124 | 0.508 | 0.053 | 0.775 | -0.096 | 0.609 | **0.449** | **0.011** | 0.077 | 0.680 | 0.092 | 0.624 | 0.007 | 0.970 | 0.136 | 0.465 | 0.163 | 0.409 | -0.018 | 0.921 | -0.242 | 0.199 | 0.043 | 0.819 | 0.167 | 0.369 | 0.116 | 0.535 |

**Supplemental Table 14.** Spearman’s correlation coefficients (r) and p-values for associations between baseline clinical scores and ^11^C-UCB-J V_T_ in the Parkinson’s disease (PD) group (n = 31), where idiopathic PD (n = 26) and LRRK2-PD (n = 5) cases were included. Note that University of Pennsylvania Smell Identification Test (UPSIT) scores were not collected for three participants, and Apathy Evaluation Scale (AES) score was not collected for one participant. Age and sex were included as covariates. Statistically significant results (p<0.05) are highlighted with bold font.

|  | **Total MDS-UPDRS** | | **MDS-UPDRS**  **III** | | **SCOPA-AUT** | | **PDSS** | | **ESS** | | **RBDSQ** | | **MCAS** | | **PFS-16** | | **KPPS** | | **UPSIT** | | **BDI** | | **AES** | | **MoCA** | | **SDMT** | | **LNSI** | |
| --- | --- | --- | --- | --- | --- | --- | --- | --- | --- | --- | --- | --- | --- | --- | --- | --- | --- | --- | --- | --- | --- | --- | --- | --- | --- | --- | --- | --- | --- | --- |
|  | **r** | ***p*** | **r** | ***p*** | **r** | ***p*** | **r** | ***p*** | **r** | ***p*** | **r** | ***p*** | **r** | ***p*** | **r** | ***p*** | **r** | ***p*** | **r** | ***p*** | **r** | ***p*** | **r** | ***p*** | **r** | ***p*** | **r** | ***p*** | **r** | ***p*** |
| **Occipital lobe** | -0.150 | 0.437 | -0.223 | 0.244 | 0.235 | 0.220 | -0.035 | 0.858 | -0.213 | 0.268 | **0.459** | **0.012** | 0.278 | 0.144 | 0.120 | 0.534 | -0.006 | 0.974 | 0.285 | 0.158 | -0.058 | 0.764 | 0.351 | 0.067 | 0.031 | 0.874 | -0.161 | 0.404 | -0.204 | 0.289 |
| **Insular cortex** | -0.124 | 0.522 | -0.148 | 0.443 | 0.237 | 0.216 | -0.060 | 0.756 | -0.065 | 0.738 | **0.503** | **0.005** | 0.142 | 0.463 | 0.239 | 0.211 | 0.116 | 0.548 | 0.217 | 0.286 | -0.171 | 0.374 | 0.271 | 0.163 | 0.234 | 0.221 | 0.068 | 0.725 | -0.053 | 0.784 |
| **Temporal lobe** | -0.149 | 0.441 | -0.216 | 0.261 | 0.310 | 0.102 | -0.162 | 0.400 | -0.080 | 0.678 | **0.518** | **0.004** | 0.184 | 0.340 | 0.148 | 0.443 | 0.094 | 0.626 | 0.241 | 0.236 | -0.073 | 0.707 | 0.359 | 0.060 | 0.136 | 0.481 | 0.036 | 0.855 | -0.149 | 0.439 |
| **Frontal lobe** | -0.099 | 0.610 | -0.183 | 0.342 | 0.353 | 0.061 | -0.024 | 0.903 | -0.177 | 0.357 | **0.458** | **0.013** | 0.197 | 0.305 | 0.232 | 0.225 | 0.033 | 0.865 | 0.218 | 0.285 | -0.128 | 0.508 | 0.209 | 0.286 | 0.076 | 0.694 | -0.142 | 0.463 | -0.207 | 0.282 |
| **Parietal lobe** | -0.196 | 0.308 | -0.287 | 0.131 | 0.246 | 0.199 | 0.035 | 0.855 | -0.231 | 0.228 | **0.392** | **0.036** | 0.253 | 0.185 | 0.122 | 0.529 | 0.006 | 0.974 | 0.266 | 0.188 | -0.066 | 0.734 | 0.319 | 0.098 | -0.001 | 0.996 | -0.184 | 0.340 | -0.174 | 0.367 |
| **Thalamus** | -0.210 | 0.275 | -0.302 | 0.112 | 0.258 | 0.177 | -0.066 | 0.735 | -0.100 | 0.605 | **0.514** | **0.004** | 0.137 | 0.480 | 0.244 | 0.202 | 0.134 | 0.488 | 0.203 | 0.320 | -0.094 | 0.628 | 0.362 | 0.058 | 0.230 | 0.229 | -0.083 | 0.669 | -0.015 | 0.938 |
| **Posterior cingulate** | -0.153 | 0.429 | -0.216 | 0.260 | 0.226 | 0.238 | 0.010 | 0.960 | -0.151 | 0.435 | **0.504** | **0.005** | 0.307 | 0.105 | 0.111 | 0.568 | 0.047 | 0.810 | 0.274 | 0.176 | -0.066 | 0.735 | 0.217 | 0.267 | 0.024 | 0.901 | -0.056 | 0.774 | -0.130 | 0.500 |
| **Anterior cingulate** | -0.128 | 0.509 | -0.192 | 0.318 | 0.361 | 0.054 | 0.017 | 0.931 | -0.129 | 0.506 | **0.501** | **0.006** | 0.166 | 0.389 | 0.292 | 0.125 | 0.107 | 0.581 | 0.208 | 0.307 | -0.163 | 0.398 | 0.168 | 0.392 | 0.202 | 0.292 | -0.029 | 0.881 | -0.077 | 0.693 |
| **Substantia nigra** | -0.040 | 0.836 | -0.086 | 0.658 | **0.378** | **0.043** | -0.143 | 0.461 | 0.045 | 0.819 | 0.343 | 0.068 | 0.327 | 0.083 | 0.278 | 0.144 | 0.136 | 0.481 | 0.147 | 0.474 | 0.230 | 0.231 | 0.253 | 0.193 | -0.059 | 0.761 | 0.187 | 0.331 | -0.214 | 0.265 |
| **Caudate** | -0.135 | 0.485 | -0.312 | 0.100 | **0.397** | **0.033** | -0.188 | 0.329 | -0.054 | 0.781 | **0.423** | **0.022** | 0.045 | 0.818 | 0.290 | 0.127 | 0.302 | 0.111 | 0.226 | 0.268 | -0.039 | 0.839 | 0.300 | 0.121 | 0.268 | 0.159 | -0.109 | 0.575 | -0.033 | 0.865 |
| **Putamen** | -0.208 | 0.280 | -0.332 | 0.079 | 0.297 | 0.118 | -0.094 | 0.626 | 0.088 | 0.648 | **0.504** | **0.005** | 0.160 | 0.406 | 0.206 | 0.283 | 0.095 | 0.625 | 0.219 | 0.283 | -0.024 | 0.900 | 0.259 | 0.183 | 0.162 | 0.400 | 0.065 | 0.739 | -0.143 | 0.461 |

**Supplemental Table 15.** Spearman’s correlation coefficients (r) and p-values for associations between baseline clinical scores and ^11^C-UCB-J BP_ND_ in the Parkinson’s disease (PD) group (n = 33), where idiopathic PD (n = 27) and LRRK2-PD (n = 6) cases were included. Note that University of Pennsylvania Smell Identification Test (UPSIT) scores were not collected for three participants, and Apathy Evaluation Scale (AES) score was not collected for one participant. Age and sex were included as covariates. Statistically significant results (p<0.05) are highlighted with bold font.

|  | **Total MDS-UPDRS** | | **MDS-UPDRS**  **III** | | **SCOPA-AUT** | | **PDSS** | | **ESS** | | **RBDSQ** | | **MCAS** | | **PFS-16** | | **KPPS** | | **UPSIT** | | **BDI** | | **AES** | | **MoCA** | | **SDMT** | | **LNSI** | |
| --- | --- | --- | --- | --- | --- | --- | --- | --- | --- | --- | --- | --- | --- | --- | --- | --- | --- | --- | --- | --- | --- | --- | --- | --- | --- | --- | --- | --- | --- | --- |
|  | **r** | ***p*** | **r** | ***p*** | **r** | ***p*** | **r** | ***p*** | **r** | ***p*** | **r** | ***p*** | **r** | ***p*** | **r** | ***p*** | **r** | ***p*** | **r** | ***p*** | **r** | ***p*** | **r** | ***p*** | **r** | ***p*** | **r** | ***p*** | **r** | ***p*** |
| **Occipital lobe** | -0.192 | 0.301 | -0.320 | 0.079 | 0.198 | 0.285 | 0.045 | 0.811 | -0.184 | 0.321 | -0.032 | 0.863 | -0.187 | 0.313 | -0.054 | 0.771 | 0.019 | 0.917 | 0.185 | 0.347 | -0.276 | 0.133 | 0.169 | 0.373 | **0.453** | **0.011** | -0.189 | 0.307 | 0.050 | 0.791 |
| **Insular cortex** | -0.171 | 0.358 | -0.317 | 0.082 | 0.279 | 0.128 | 0.095 | 0.610 | 0.015 | 0.935 | 0.043 | 0.819 | -0.193 | 0.299 | 0.034 | 0.856 | 0.119 | 0.525 | 0.141 | 0.474 | -0.256 | 0.164 | 0.077 | 0.688 | **0.577** | **<0.001** | -0.063 | 0.737 | -0.003 | 0.989 |
| **Temporal lobe** | -0.183 | 0.325 | -0.333 | 0.067 | **0.359** | **0.047** | 0.113 | 0.545 | -0.099 | 0.596 | 0.064 | 0.733 | -0.141 | 0.450 | -0.004 | 0.981 | 0.121 | 0.516 | 0.187 | 0.341 | -0.299 | 0.102 | 0.164 | 0.385 | **0.441** | **0.013** | -0.161 | 0.388 | -0.089 | 0.633 |
| **Frontal lobe** | -0.158 | 0.397 | -0.324 | 0.076 | 0.343 | 0.059 | 0.133 | 0.476 | -0.033 | 0.859 | 0.063 | 0.737 | -0.119 | 0.524 | 0.082 | 0.660 | 0.189 | 0.309 | 0.055 | 0.783 | -0.179 | 0.334 | 0.085 | 0.656 | 0.354 | 0.051 | -0.247 | 0.180 | -0.057 | 0.760 |
| **Parietal lobe** | -0.232 | 0.209 | **-0.398** | **0.026** | 0.226 | 0.222 | 0.153 | 0.412 | -0.154 | 0.407 | -0.002 | 0.989 | -0.164 | 0.379 | -0.069 | 0.712 | -0.008 | 0.966 | 0.118 | 0.549 | -0.265 | 0.150 | 0.122 | 0.522 | 0.333 | 0.067 | -0.254 | 0.169 | 0.040 | 0.829 |
| **Thalamus** | -0.194 | 0.295 | -0.354 | 0.050 | 0.181 | 0.330 | -0.011 | 0.954 | -0.021 | 0.910 | -0.002 | 0.992 | -0.231 | 0.212 | 0.011 | 0.953 | 0.144 | 0.441 | 0.044 | 0.824 | -0.088 | 0.638 | 0.143 | 0.449 | **0.398** | **0.027** | -0.195 | 0.294 | 0.077 | 0.680 |
| **Posterior cingulate** | -0.222 | 0.231 | **-0.396** | **0.027** | 0.281 | 0.125 | 0.148 | 0.426 | -0.123 | 0.511 | 0.018 | 0.921 | -0.124 | 0.505 | 0.012 | 0.951 | 0.096 | 0.607 | 0.146 | 0.457 | -0.264 | 0.151 | 0.094 | 0.621 | **0.467** | **0.008** | -0.181 | 0.331 | 0.022 | 0.905 |
| **Anterior cingulate** | -0.167 | 0.370 | -0.333 | 0.067 | 0.337 | 0.064 | 0.179 | 0.335 | -0.035 | 0.852 | 0.041 | 0.826 | -0.126 | 0.501 | 0.075 | 0.689 | 0.116 | 0.535 | 0.085 | 0.666 | -0.250 | 0.174 | 0.037 | 0.845 | **0.495** | **0.005** | -0.162 | 0.382 | -0.050 | 0.788 |
| **Substantia nigra** | -0.003 | 0.988 | -0.111 | 0.553 | **0.628** | **<0.001** | -0.129 | 0.488 | 0.056 | 0.765 | 0.105 | 0.575 | 0.146 | 0.433 | 0.292 | 0.111 | **0.416** | **0.020** | -0.107 | 0.589 | 0.124 | 0.505 | 0.207 | 0.272 | 0.272 | 0.139 | -0.016 | 0.931 | -0.179 | 0.335 |
| **Caudate** | -0.153 | 0.412 | -0.340 | 0.061 | 0.285 | 0.120 | -0.081 | 0.667 | -0.005 | 0.978 | 0.109 | 0.559 | -0.148 | 0.426 | 0.100 | 0.592 | 0.268 | 0.145 | -0.027 | 0.892 | -0.065 | 0.730 | 0.098 | 0.607 | **0.370** | **0.040** | -0.192 | 0.301 | 0.040 | 0.830 |
| **Putamen** | -0.235 | 0.204 | **-0.424** | **0.017** | 0.321 | 0.078 | 0.158 | 0.395 | 0.088 | 0.638 | 0.157 | 0.398 | -0.148 | 0.428 | 0.087 | 0.643 | 0.197 | 0.287 | 0.020 | 0.920 | -0.221 | 0.231 | 0.084 | 0.659 | **0.476** | **0.007** | -0.097 | 0.603 | -0.028 | 0.879 |

**Supplemental Table 16.** Linear mixed-effects model results for longitudinal changes in regional grey matter volumes. β coefficients reflect the fixed-effect parameter estimates (based on t-tests), while the accompanying F- and p-values are drawn from the Type III Tests of Fixed Effects. Models examined the main effects of group (healthy controls [HC], n = 22; idiopathic Parkinson’s disease [iPD], n = 20), time (modelled continuously as days since baseline), and the group × time interaction, with age at baseline and sex entered as covariates. Statistically significant effects (p < 0.05) appear in bold.

|  |  | **Time** | | | **Group** | | | **Time x Group** | | |
| --- | --- | --- | --- | --- | --- | --- | --- | --- | --- | --- |
|  | | $\boldsymbol{\beta}$ | **F** | ***p*** | $\boldsymbol{\beta}$ | **F** | ***p*** | $\boldsymbol{\beta}$ | **F** | ***p*** |
| **Occipital lobe** | | **-2.864** | **33.540** | **<0.001** | 2131.078 | 0.938 | 0.337 | 1.258 | 2.656 | 0.111 |
| **Insular cortex** | | **-0.155** | **28.426** | **<0.001** | -3.102 | 0.000 | 0.991 | -0.021 | 0.118 | 0.733 |
| **Temporal lobe** | | **-3.446** | **36.812** | **<0.001** | 3106.088 | 1.232 | 0.272 | 1.558 | 3.140 | 0.084 |
| **Frontal lobe** | | **-7.108** | **103.759** | **<0.001** | 1548.323 | 0.121 | 0.730 | 1.342 | 1.128 | 0.295 |
| **Parietal lobe** | | **-3.943** | **50.326** | **<0.001** | 4324.440 | 2.608 | 0.112 | 1.318 | 2.026 | 0.162 |
| **Thalamus** | | 0.037 | 0.690 | 0.411 | 210.141 | 0.478 | 0.492 | -0.318 | 1.166 | 0.287 |
| **Posterior cingulate** | | **-0.202** | **24.536** | **<0.001** | 247.646 | 0.786 | 0.379 | 0.053 | 0.549 | 0.463 |
| **Anterior cingulate** | | **-1.081** | **112.831** | **<0.001** | -161.050 | 0.037 | 0.849 | 0.047 | 0.055 | 0.816 |
| **Substantia nigra** | | 0.002 | 0.153 | 0.698 | -5.051 | 1.277 | 0.263 | -0.001 | 0.095 | 0.760 |
| **Caudate** | | **-0.084** | **27.437** | **<0.001** | 213.895 | 1.990 | 0.164 | 0.004 | 0.020 | 0.889 |
| **Putamen** | | **-0.122** | **16.696** | **<0.001** | 240.082 | 1.593 | 0.212 | -0.184 | 3.094 | 0.086 |

**Supplemental Table 17.** Linear mixed-effects model results for longitudinal changes in DAT SPECT standardised uptake value ratios (SUVRs). β coefficients reflect the fixed-effect parameter estimates (based on t-tests), while the accompanying F- and p-values are drawn from the Type III Tests of Fixed Effects. Models examined the main effects of group (healthy controls [HC], n = 17; idiopathic Parkinson’s disease [iPD], n = 20), time (modelled continuously as days since baseline), and the group × time interaction, with age at baseline and sex entered as covariates. Statistically significant effects (p < 0.05) appear in bold.

|  |  | **Time** | | | **Group** | | | **Time x Group** | | |
| --- | --- | --- | --- | --- | --- | --- | --- | --- | --- | --- |
|  | | $\boldsymbol{\beta}$ | **F** | ***p*** | $\boldsymbol{\beta}$ | **F** | ***p*** | $\boldsymbol{\beta}$ | **F** | ***p*** |
| **Caudate** | | -2.69x10^-4^ | 1.769 | 0.192 | **1.356** | **98.232** | **<0.001** | 3.24x10^-4^ | 4.077 | 0.051 |
| **Putamen** | | 1.45x10^-4^ | 0.116 | 0.735 | **1.785** | **206.539** | **<0.001** | 2.34x10^-4^ | 2.035 | 0.162 |
| **Striatum** | | -1.94x10^-4^ | 0.585 | 0.449 | **1.556** | **145.502** | **<0.001** | 2.69x10^-4^ | 3.004 | 0.091 |

**Supplemental Table 18.** Linear mixed-effects model results for longitudinal changes in ¹¹C-DASB BP_ND_. β coefficients reflect the fixed-effect parameter estimates (based on t-tests), while the accompanying F- and p-values are drawn from the Type III Tests of Fixed Effects. Models examined the main effects of group (healthy controls [HC], n = 22; idiopathic Parkinson’s disease [iPD], n = 20), time (modelled continuously as days since baseline), and the group × time interaction, with age at baseline and sex entered as covariates. Statistically significant effects (p < 0.05) appear in bold.

|  |  | **Time** | | | **Group** | | | **Time x Group** | | |
| --- | --- | --- | --- | --- | --- | --- | --- | --- | --- | --- |
|  | | $\boldsymbol{\beta}$ | **F** | ***p*** | $\boldsymbol{\beta}$ | **F** | ***p*** | $\boldsymbol{\beta}$ | **F** | ***p*** |
| **Occipital lobe** | | -3.19x10^-6^ | 0.181 | 0.673 | 0.039 | 1.933 | 0.169 | -1.33x10^-5^ | 0.068 | 0.796 |
| **Insular cortex** | | 1.08x10^-5^ | 0.875 | 0.355 | **0.098** | **9.508** | **0.003** | -5.68x10^-5^ | 1.888 | 0.176 |
| **Temporal lobe** | | 2.98x10^-5^ | 0.760 | 0.389 | 0.042 | 2.445 | 0.123 | 9.29x10^-7^ | 0.670 | 0.418 |
| **Frontal lobe** | | 2.32x10^-5^ | 0.005 | 0.944 | 0.053 | 2.654 | 0.107 | -1.88x10^-5^ | 0.440 | 0.510 |
| **Parietal lobe** | | -2.44x10^-7^ | 0.716 | 0.402 | **0.079** | **6.822** | **0.011** | -4.62x10^-5^ | 0.701 | 0.407 |
| **Thalamus** | | 2.86x10^-5^ | 0.298 | 0.588 | 0.049 | 0.219 | 0.641 | 8.91x10^-5^ | 0.079 | 0.781 |
| **Posterior cingulate** | | -2.48x10^-5^ | 1.420 | 0.239 | 0.060 | 2.699 | 0.104 | -5.20x10^-5^ | 0.179 | 0.674 |
| **Anterior cingulate** | | 1.01x10^-5^ | 0.020 | 0.889 | 0.043 | 1.308 | 0.257 | -1.83x10^-5^ | 0.238 | 0.629 |
| **Substantia nigra** | | -1.25x10^-4^ | 0.183 | 0.670 | 0.146 | 0.760 | 0.386 | -3.87x10^-5^ | 0.051 | 0.822 |
| **Caudate** | | -4.02x10^-5^ | 0.340 | 0.563 | **0.201** | **12.786** | **<0.001** | -1.30x10^-5^ | 0.089 | 0.767 |
| **Putamen** | | 6.28x10^-5^ | 1.084 | 0.303 | **0.224** | **10.716** | **0.002** | 3.97x10^-5^ | 0.055 | 0.816 |

**Supplemental Table 19.** Linear mixed-effects model results for longitudinal changes in ^11^C-UCB-J V_T_. β coefficients reflect the fixed-effect parameter estimates (based on t-tests), while the accompanying F- and p-values are drawn from the Type III Tests of Fixed Effects. Models examined the main effects of group (healthy controls [HC], n = 18; idiopathic Parkinson’s disease [iPD], n = 18), time (modelled continuously as days since baseline), and the group × time interaction, with age at baseline and sex entered as covariates. Statistically significant effects (p < 0.05) appear in bold.

|  |  | **Time** | | | **Group** | | | **Time x Group** | | |
| --- | --- | --- | --- | --- | --- | --- | --- | --- | --- | --- |
|  | | $\boldsymbol{\beta}$ | **F** | ***p*** | $\boldsymbol{\beta}$ | **F** | ***p*** | $\boldsymbol{\beta}$ | **F** | ***p*** |
| **Occipital lobe** | | 2.62x10^-4^ | 0.484 | 0.490 | 0.172 | 0.084 | 0.773 | -0.001 | 1.280 | 0.263 |
| **Insular cortex** | | 0.001 | 0.005 | 0.945 | -0.198 | 0.075 | 0.785 | -0.001 | 0.585 | 0.448 |
| **Temporal lobe** | | 0.001 | 0.001 | 0.976 | 0.065 | 0.010 | 0.920 | -0.001 | 0.788 | 0.379 |
| **Frontal lobe** | | 3.05x10^-4^ | <0.001 | 0.983 | -0.110 | 0.032 | 0.859 | -0.001 | 0.259 | 0.613 |
| **Parietal lobe** | | 1.57x10^-4^ | 0.535 | 0.468 | 0.412 | 0.467 | 0.496 | -0.001 | 0.981 | 0.327 |
| **Thalamus** | | -1.84x10^-4^ | 0.836 | 0.365 | 0.373 | 0.596 | 0.442 | -4.91x10^-4^ | 0.272 | 0.604 |
| **Posterior cingulate** | | 1.43x10^-4^ | 0.240 | 0.626 | 0.076 | 0.014 | 0.905 | -0.001 | 0.496 | 0.485 |
| **Anterior cingulate** | | 4.70x10^-4^ | 0.004 | 0.947 | -0.218 | 0.095 | 0.758 | -0.001 | 0.501 | 0.482 |
| **Substantia nigra** | | -1.30x10^-5^ | 0.080 | 0.778 | 0.514 | 2.715 | 0.104 | -1.45x10^-4^ | 0.057 | 0.812 |
| **Caudate** | | 2.38x10^-4^ | 0.124 | 0.727 | 1.096 | 3.093 | 0.083 | -0.001 | 0.592 | 0.445 |
| **Putamen** | | 0.001 | 0.058 | 0.811 | 0.457 | 0.320 | 0.573 | -0.001 | 0.239 | 0.627 |

**Supplemental Table 20.** Linear mixed-effects model results for longitudinal changes in ^11^C-UCB-J BP_ND_. β coefficients reflect the fixed-effect parameter estimates (based on t-tests), while the accompanying F- and p-values are drawn from the Type III Tests of Fixed Effects. Models examined the main effects of group (healthy controls [HC], n = 21; idiopathic Parkinson’s disease [iPD], n = 20), time (modelled continuously as days since baseline), and the group × time interaction, with age at baseline and sex entered as covariates. Statistically significant effects (p < 0.05) appear in bold.

|  |  | **Time** | | | **Group** | | | **Time x Group** | | |
| --- | --- | --- | --- | --- | --- | --- | --- | --- | --- | --- |
|  | | $\boldsymbol{\beta}$ | **F** | ***p*** | $\boldsymbol{\beta}$ | **F** | ***p*** | $\boldsymbol{\beta}$ | **F** | ***p*** |
| **Occipital lobe** | | 1.54x10^-4^ | 0.006 | 0.937 | 0.048 | 0.157 | 0.693 | -2.87x10^-4^ | 1.230 | 0.272 |
| **Insular cortex** | | 2.20x10^-4^ | <0.001 | 0.994 | 0.015 | 0.009 | 0.926 | -4.42x10^-4^ | 1.629 | 0.207 |
| **Temporal lobe** | | 1.76x10^-4^ | 0.001 | 0.972 | 0.036 | 0.063 | 0.802 | -3.41x10^-4^ | 1.176 | 0.283 |
| **Frontal lobe** | | 1.67x10^-4^ | 0.146 | 0.704 | -0.005 | 0.002 | 0.969 | -2.29x10^-4^ | 0.675 | 0.415 |
| **Parietal lobe** | | 8.67x10^-5^ | 0.049 | 0.826 | 0.058 | 0.220 | 0.640 | -2.32x10^-4^ | 0.754 | 0.389 |
| **Thalamus** | | -3.49x10^-5^ | 0.149 | 0.702 | 0.034 | 0.097 | 0.757 | -3.00x10^-6^ | <0.001 | 0.987 |
| **Posterior cingulate** | | 1.04x10^-4^ | 0.002 | 0.963 | -0.015 | 0.014 | 0.907 | -2.22x10^-4^ | 0.623 | 0.433 |
| **Anterior cingulate** | | 1.86x10^-4^ | 0.005 | 0.943 | -0.020 | 0.017 | 0.898 | -3.47x10^-4^ | 1.061 | 0.308 |
| **Substantia nigra** | | -3.84x10^-5^ | 0.400 | 0.530 | 0.115 | 3.170 | 0.078 | -1.45x10^-5^ | 0.010 | 0.920 |
| **Caudate** | | 1.76x10^-4^ | 0.001 | 0.970 | 0.213 | 2.484 | 0.119 | -3.43x10^-4^ | 1.877 | 0.177 |
| **Putamen** | | 7.79x10^-6^ | 0.005 | 0.946 | 0.084 | 0.274 | 0.602 | -3.86x10^-5^ | 0.013 | 0.910 |
